# Circadian Clock Genes Contribute to the Regulation of Hair Follicle Cycling

**DOI:** 10.1371/journal.pgen.1000573

**Published:** 2009-07-24

**Authors:** Kevin K. Lin, Vivek Kumar, Mikhail Geyfman, Darya Chudova, Alexander T. Ihler, Padhraic Smyth, Ralf Paus, Joseph S. Takahashi, Bogi Andersen

**Affiliations:** 1 Department of Biological Chemistry, University of California Irvine, Irvine, California, United States of America; 2 Department of Medicine, University of California Irvine, Irvine, California, United States of America; 3 Institute for Genomics and Bioinformatics, University of California Irvine, Irvine, California, United States of America; 4 Howard Hughes Medical Institute, Northwestern University, Evanston, Illinois, United States of America; 5 Department of Neurobiology and Physiology, Northwestern University, Evanston, Illinois, United States of America; 6 Department of Neuroscience, University of Texas Southwestern Medical Center, Dallas, Texas, United States of America; 7 Department of Computer Science, University of California Irvine, Irvine, California, United States of America; 8 Department of Dermatology, University of Luebeck, Luebeck, Germany; 9 School of Translational Medicine, University of Manchester, Manchester, United Kingdom; University of Pennsylvania School of Medicine, United States of America

## Abstract

Hair follicles undergo recurrent cycling of controlled growth (anagen), regression (catagen), and relative quiescence (telogen) with a defined periodicity. Taking a genomics approach to study gene expression during synchronized mouse hair follicle cycling, we discovered that, in addition to circadian fluctuation, CLOCK–regulated genes are also modulated in phase with the hair growth cycle. During telogen and early anagen, circadian clock genes are prominently expressed in the secondary hair germ, which contains precursor cells for the growing follicle. Analysis of *Clock* and *Bmal1* mutant mice reveals a delay in anagen progression, and the secondary hair germ cells show decreased levels of phosphorylated Rb and lack mitotic cells, suggesting that circadian clock genes regulate anagen progression via their effect on the cell cycle. Consistent with a block at the G1 phase of the cell cycle, we show a significant upregulation of p21 in *Bmal1* mutant skin. While circadian clock mechanisms have been implicated in a variety of diurnal biological processes, our findings indicate that circadian clock genes may be utilized to modulate the progression of non-diurnal cyclic processes.

## Author Summary

The hair follicle renews itself by repeatedly cycling among growth, regression, and rest phases. One function of hair follicle cycling is to allow seasonal changes in hair growth. Understanding the regulation of hair follicle cycling is also of interest because abnormal regulation of hair cycle control genes is responsible for several types of human hair growth disorders and skin cancers. We report here that *Clock* and *Bmal1* genes, which control circadian rhythms, are also important for the regulation of hair follicle cycling, a biological process of much longer duration than 24 hours. Detailed analysis of skin from mice mutated for central clock genes indicates a significant delay in the progression of the hair growth phase. We show that clock genes affect the expression of key cell cycle control genes and that keratinocytes in a critical compartment of the hair follicles in *Bmal1* mutant mice are halted in the G1 phase of the cell cycle. These findings provide novel insight into circadian control mechanisms in modulating the progression of cyclic biological processes on different time scales.

## Introduction

Evolutionarily conserved hair follicle cycling is thought to provide mechanisms for controlling the length of hair in specific body sites, and to allow the periodic shedding of fur in response to seasonal changes in mammals [1]. The periodicity of the hair growth cycle ranges from approximately three-weeks in synchronized hair follicles of mouse dorsal skin to several years in hair follicles of human scalp where the follicles undergo an extended period of hair growth [2]. In mice, hair follicle morphogenesis is completed around postnatal day (P) 14, at which time the follicle enters a phase called catagen. During catagen, extensive apoptosis in the lower two-thirds of the follicle results in its dramatic regression, leaving intact the stem cell-containing bulge region. The hair follicle then goes through a relative quiescent phase referred to as telogen. Following telogen, the stem cells become activated, likely in response to inductive signals from the dermal papilla, and the follicle enters the growth phase characterized by active keratinocyte proliferation and differentiation known as anagen. During the first two natural hair growth cycles in mice, the follicles of the dorsal skin are synchronized in progressing through the cycle, allowing the study of the mechanisms of natural hair follicle cycling. In addition, tightly synchronized hair growth cycle can be initiated by depilation of hair shafts (e.g., waxing) during the telogen phase, with the caveat, however, of triggering an injury response [3]. Although components of numerous molecular pathways, including TGFβ/BMP family members and their antagonists, FGFs and steroid hormone receptors, have been implicated in the control of hair follicle cycling [1], [4]–[8], the underlying mechanisms regulating its timing remain elusive [9].

The periodicity of the hair growth cycle is reminiscent of other cyclic processes, such as the circadian rhythm where distinct clock mechanisms exist. The regulation of circadian rhythm consists of positive and negative regulatory feedback loops, having a period of approximately 24 hours [10]–[12]. The positive feedback loop is regulated by two DNA-binding basic helix-loop-helix (bHLH) PAS domain transcription factors CLOCK and BMAL1 [13], which form a heterodimer and transcriptionally activate genes containing E-boxes in their regulatory regions. Among their targets are PER (PER 1, 2 and 3) and CRY (CRY1 and 2) that form heterodimers and repress their own transcription via direct interactions with CLOCK/BMAL1 [14]. Additional targets include RORs (*Rora*, *Rorb*, and *Rorc*) and REV-ERBs (*Nr1d1* and *Nr1d2*), which are members of a subfamily of orphan nuclear receptors that bind to ROR response elements (RREs) to transcriptionally activate and repress *Bmal1*, respectively [15],[16]. Other CLOCK-controlled targets, such as the transcription factors DBP and TEF, are not part of the central circadian mechanism, but are thought to mediate many of its physiological effects [17],[18].

In mammals, the central pacemaker that controls circadian behavior is in the suprachiasmatic nucleus (SCN) of the hypothalamus. It is now widely accepted that the circadian control mechanisms are not limited to the SCN, but instead, the molecular clock is expressed and operative in most peripheral tissues [19]–[21]. Increasing evidence highlights the importance for the SCN in coordinating the independent oscillation of molecular clocks in peripheral tissues [10]. Although several studies demonstrated circadian rhythm within human and mouse skin as well as oral keratinocytes [22]–[26], a functional role for the circadian clock genes in skin has yet to be determined. Taking a genomics approach to study gene expression during synchronized mouse hair follicle cycling, we discovered that the genes which control circadian rhythm are utilized to modulate the progression of hair follicle cycling, a biological process of much longer duration than the diurnal period. We found a significant delay in anagen progression in both *Clock* and *Bmal1* mutant mice, and the secondary hair germ cells within mutant hair follicles show decreased levels of phosphorylated Rb and lack mitotic cells, suggesting the circadian clock modulate anagen progression via its effect on the cell cycle.

## Results

### Identification of Periodically Expressed Genes during Hair Follicle Cycling from Time-Course Expression Profiles

**Figure 1:**
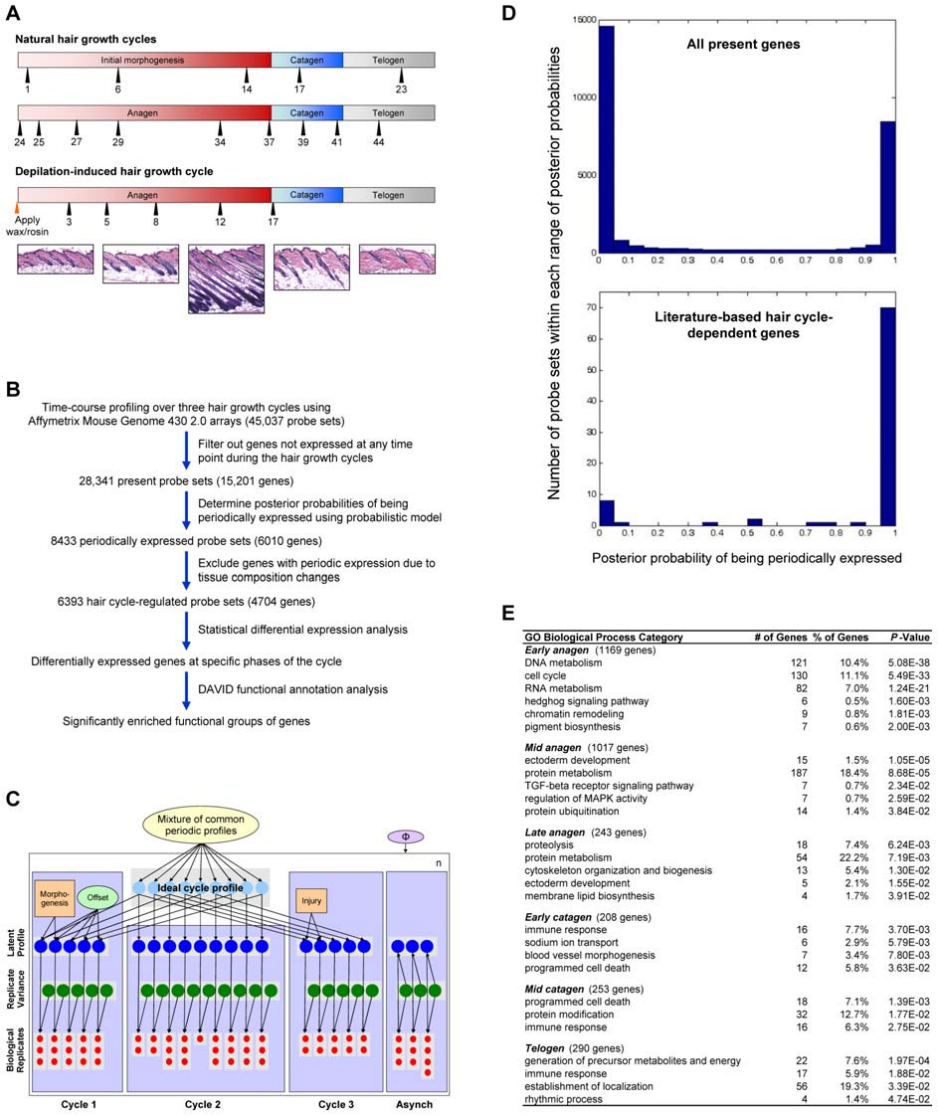
Identification of hair cycle–regulated genes using probabilistic models. (A) Representative time points selected for gene expression profiling of the natural and depilation-induced hair growth cycles. Time points for natural cycles represent postnatal days, and time points for depilation-induced cycle indicate the number of days following depilation. Since the three cycles have different duration, the schematic timeline is not in actual time scale. Bottom panel consists of representative histology of dorsal skin at key phases of synchronized hair follicle cycling. (B) Overview of data processing and statistical analyses. Note that samples for the first synchronized hair growth cycle and asynchronous time points were profiled in an earlier study using an older generation array (Affymetrix Murine Genome U74Av2). (C) Schematic of the probabilistic model for detection of periodic gene expression changes during hair follicle cycling. See Materials and Methods for a detailed description of the schematic. Asynch – asynchronous cycle. (D) Histogram of the number of probe sets within each range of the indicated posterior probabilities of being periodically expressed. Top panel is the histogram of probabilities for all present genes during the hair growth cycle. The bottom panel is the histogram of probabilities for the literature-based hair cycle-dependent genes. (E) List of significantly enriched GO Biological Process categories within the sets of genes specifically upregulated at the indicated phases of the hair growth cycle. Due to the redundancy of categories, not all are listed. The number of genes upregulated at each phase of the cycle is in parentheses. Statistical enrichment of each category is shown as *P*-values calculated using a modified Fisher Exact test (DAVID functional annotation analysis).

To investigate the molecular control of hair follicle cycling, we profiled mRNA expression in mouse dorsal skin at multiple time points in the synchronized second postnatal hair growth cycle and in a depilation-induced hair growth cycle. By combining this data with a study profiling the postnatal completion of hair follicle morphogenesis as well as the first catagen and telogen [27], we obtained genome-wide time-course expression profiles for three distinct hair growth cycles (Figure 1A and 1B). To detect genes with periodic expression changes over multiple hair growth cycles, we constructed a probabilistic model with periodic and background components (graphical model shown in Figure 1C). The periodic component includes a mixture model of shared periodic expression profiles and allows deviation from perfect periodicity due to initial hair follicle morphogenesis or injury response to depilation. Given this model structure and observed data, we used Bayesian inference techniques to infer periodic profiles and posterior probability of periodicity for each of the probe sets [28] (Text S1).

Applying this model to the 28,341 expressed probe sets, we found that 8433 probe sets have posterior probability of greater than 0.95 of being periodically expressed (Figure 1D, top panel). Probe sets with periodic profiles over the hair growth cycle were further narrowed to 6393 probe sets exhibiting periodic gene expression changes that cannot be explained by changes in tissue composition (Figure S1); we define this set of genes as hair cycle-regulated genes (Figure 1B and Table S1). To assess the sensitivity of the calculated posterior probabilities, we compiled a list of literature-based hair cycle-dependent genes from a comprehensive unbiased literature search of genes whose expression patterns have been shown to be hair cycle-dependent using quantitative or semi-quantitative RNA methods (Table S2). We found that over 80% of literature-based hair cycle-dependent genes have posterior probabilities of greater than 0.95 (Figure 1D, bottom panel); for genes that have posterior probabilities of lower than 0.95, their expression profiles either do not visually appear to be periodic or are detected at a low intensity level. From these findings, we conclude that our probabilistic model is accurate and robust in identifying periodic expression changes during hair follicle cycling.

### Distinct Functional Groups of Genes Are Activated at Different Phases of Hair Follicle Cycling

To identify genes which are activated at specific phases of the hair growth cycle, we performed statistical differential analysis of hair cycle-regulated genes by comparing the expression from one phase to the next (e.g., genes upregulated at early anagen are significantly differentially expressed genes between telogen and early anagen samples). We then searched for significantly enriched Gene Ontology (GO) Biological Process categories within the different sets of genes upregulated at specific phases of the hair growth cycle. Among the genes upregulated at early anagen is a significantly overrepresented number of cell cycle and DNA/RNA metabolism genes, as well as other genes that are required during proliferation (Figure 1E). Many of the other enriched functional categories are also expected based on our current knowledge of hair follicle biology, validating the value of this approach. For example, members of the Hedgehog signaling pathway are upregulated during early anagen (Figure S2), consistent with their role in proliferation of keratinocytes. In contrast, TGFβ/BMP signaling pathway genes are upregulated towards the later stages of anagen, and these genes have been reported to be involved in hair follicle differentiation and apoptosis. In addition to identifying several pathways known to be involved in hair follicle regulation [1], [4]–[7], our enrichment analysis identified functional categories that were unexpected, potentially providing novel insights into regulation of hair follicle cycling. For instance, telogen is often referred to as the “quiescent phase” of the cycle, but we found significantly enriched group of genes annotated with the function of *generation of precursor metabolites and energy* (*P* = 1.97E-4) and *establishment of localization* (*P* = 3.39E-2) that are upregulated during telogen, suggesting that many active molecular processes are occurring during telogen (Figure 1E).

### CLOCK–Regulated Genes Are Periodically Expressed in Phase with the Hair Growth Cycle

**Figure 2:**
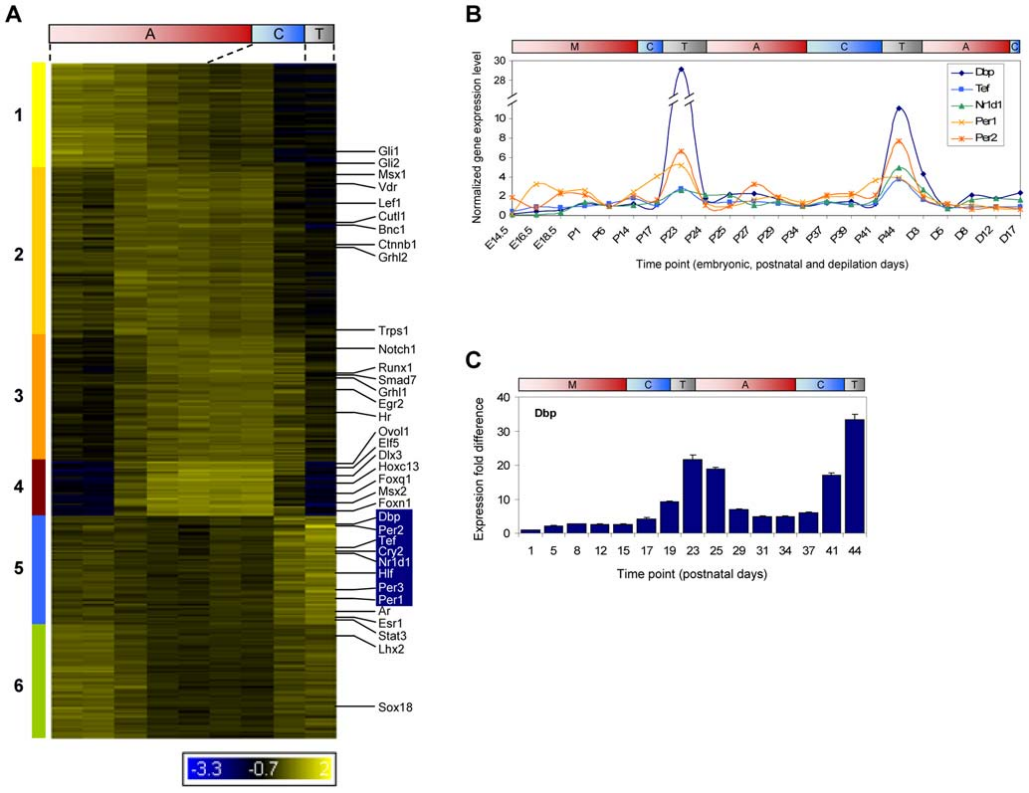
CLOCK–regulated genes are periodically expressed during hair follicle cycling. (A) Temporal clusters (labeled 1–6 and color coded) of hair cycle-regulated transcriptional regulators. Transcription factors that play key roles in hair follicle morphogenesis and/or cycling are labeled with gene symbols in black. CLOCK-regulated genes are labeled in the blue box. Expression levels are from profiling data of the second hair growth cycle and are indicated by the colorimetric ratio-scale. (B) Time-course profiles of CLOCK-controlled genes during hair follicle morphogenesis, the first two natural and depilation-induced hair growth cycles. For each gene, the expression levels were normalized relative to the lowest expressed time point of the second cycle; the first cycle (P1 to P23) was normalized separately because different array was used for profiling. Differences in magnitude of change between the first and second cycles are primarily due to differential probe set efficiencies. Note the broken y-axis. E – embryonic days; P – postnatal days; D - depilation days. (C) Q-PCR of *Dbp* using independent samples from the first two synchronized hair growth cycles. Standard deviations were determined by using three replicates normalized to *Gapdh* and fold calculated relative to the lowest expression sample. For (B–D), time points are mapped based on histology to the corresponding phases of the cycle: hair follicle morphogenesis (M), anagen (A), catagen (C), and telogen (T).

To uncover the genome-wide landscape of transcriptional regulation during hair follicle cycling, we performed time-course clustering of all hair cycle-regulated transcriptional regulators. Remarkably, this genome-wide landscape grouped together many of the key transcription factors previously shown to be functionally important during hair follicle morphogenesis and/or cycling (Figure 2A). Mice with mutations to these transcription regulators show abnormalities in hair follicle density (*Lef1*, *Msx1*, *Msx2*, *Sox18*, *Trps1*), structure (*Dlx3*, *Foxn1*, *Foxq1*, *Hoxc13*, *Notch1*, *Ovol1*, *Runx1*), morphogenesis (*Ctnnb1*, *Cutl1*, *Gli2*, *Lef1*, *Msx2*, *Smad7*), and cycling (*Hr*, *Msx2*, *Stat3*, *Vdr*).

Among the hair cycle-regulated transcriptional regulators, we were intrigued to identify a co-expressed cluster of transcription factors that are targets of circadian protein CLOCK, including *Dbp*, *Per1*, *Per2*, *Cry2*, *Nr1d1*, *Tef* and *Hlf* (Figure 2A); a hair follicle cycle-related expression of these genes has not been previously described. Correspondingly, in the set of genes significantly upregulated at telogen we also found a statistical enrichment of the GO annotation category *rhythmic processes* (Figure 1E). A time course expression profile covering hair follicle morphogenesis and the synchronized postnatal hair growth cycles shows upregulation of these CLOCK-regulated genes during telogen phases (Figure 2B). In an independent Q-PCR experiment, we confirmed up-regulation of *Dbp* around the first and second telogen, and found that the expression stays elevated during early anagen (Figure 2C). However, without a suitable DBP antibody for western/immunohistochemistry, we were unable to determine whether this upregulation of Dbp transcript levels is reflected in increased protein levels. The levels of PER2 proteins in whole skin, however, do not appear to be different between telogen and late anagen (Figure S3C). Thus, in murine skin, CLOCK-regulated genes unexpectedly exhibit periodic expression in phase with the hair growth cycle, a cyclic process with a much longer time scale than circadian rhythms.

### CLOCK–Regulated Genes Have Enhanced Circadian Expression during Telogen

**Figure 3:**
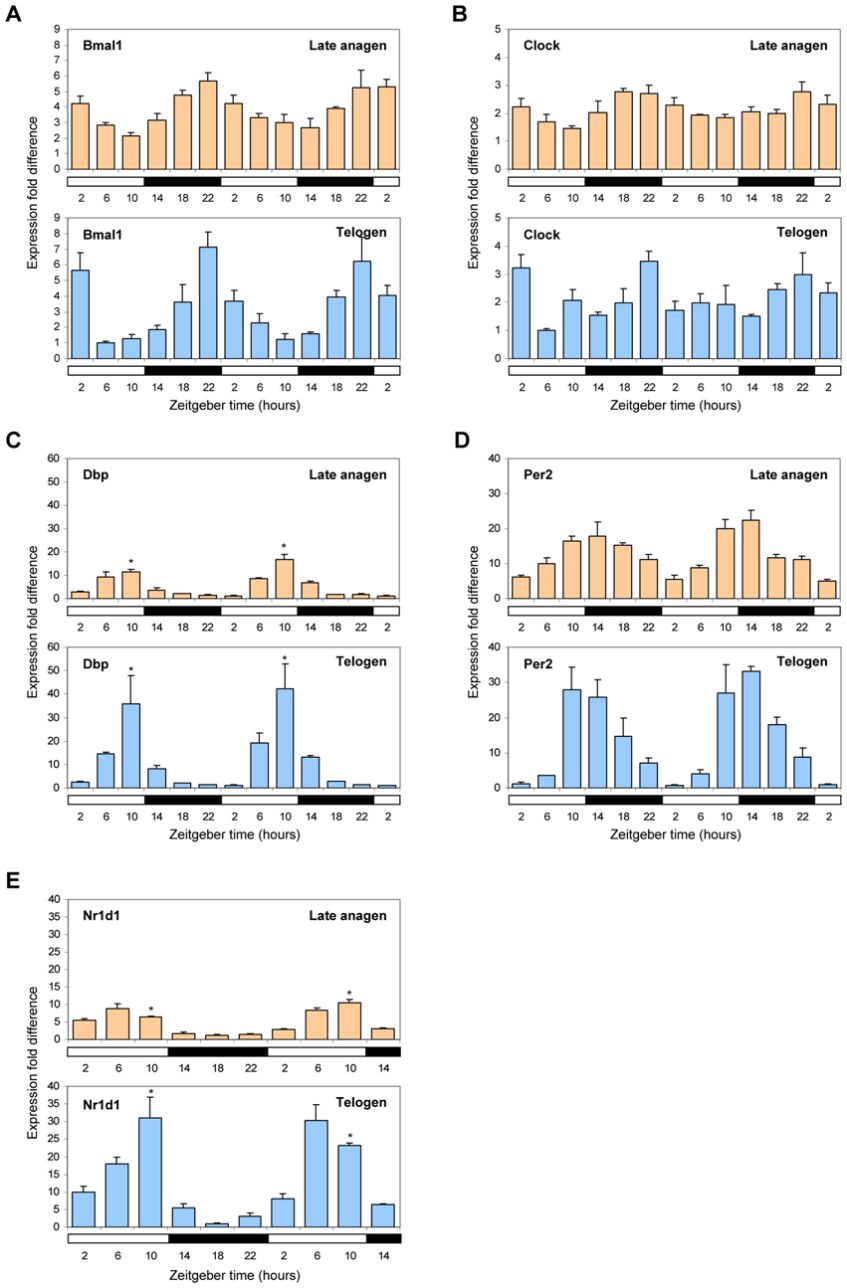
Enhanced circadian expression of CLOCK–regulated genes during telogen. Q-PCR of *Bmal1* (A), *Clock* (B), *Dbp* (C), *Per2* (D), and *Nr1d1* (E) in telogen compared to late anagen dorsal skin over the course of 48 hours (open and filled bars along the x-axis denote 12 hours light and dark phases, respectively). Expression is normalized to *Gapdh* and fold calculated relative to the lowest expression time point for both telogen and late anagen. Each error bar represents the S.E.M. for independent measurements from four mice. Asterisks denote significantly higher (*P*<0.01) expression of *Dbp* and *Nr1d1* at ZT10 in telogen.

The expression of CLOCK-regulated genes oscillates over a 24-hour period [10]–[12], while we observed a hair cycle-associated oscillation over a period of several weeks (Figure 2). Therefore, to understand the nature of the up-regulation of these genes during telogen, we performed Q-PCR for *Dbp*, *Nr1d1*, *Per2*, *Bmal1*, and *Clock* of telogen and late anagen mouse dorsal skin over the course of 48 hours of normal light/dark cycles (Figure 3). For *Dbp*, *Per2*, and *Nr1d1*, we observed circadian oscillations of expression in both telogen and late anagen, but the amplitude of the oscillation is significantly enhanced during telogen (Figure 3C–3E). The peak expression of *Dbp* and *Nr1d1* is approximately 3-fold higher (*P*<0.01) in telogen than late anagen. Similar to other peripheral tissues, such as the liver [18], we found the peak of expression for *Dbp* and *Per2* to be at Zeitgeber time (ZT) 10 and 14, respectively, where ZT0 is when light is switched on and ZT12 when light is switched off (Figure 3C and 3D). As expected, *Clock* and *Bmal1* have antiphasic circadian profiles to their target genes with peak at ZT22 (Figure 3A and 3B), suggesting that enhanced telogen expression of CLOCK-regulated genes occurs in the context of normal circadian expression profiles.

### Prominent Expression of Clock Genes in the Secondary Hair Germ during Telogen and Early Anagen

**Figure 4:**
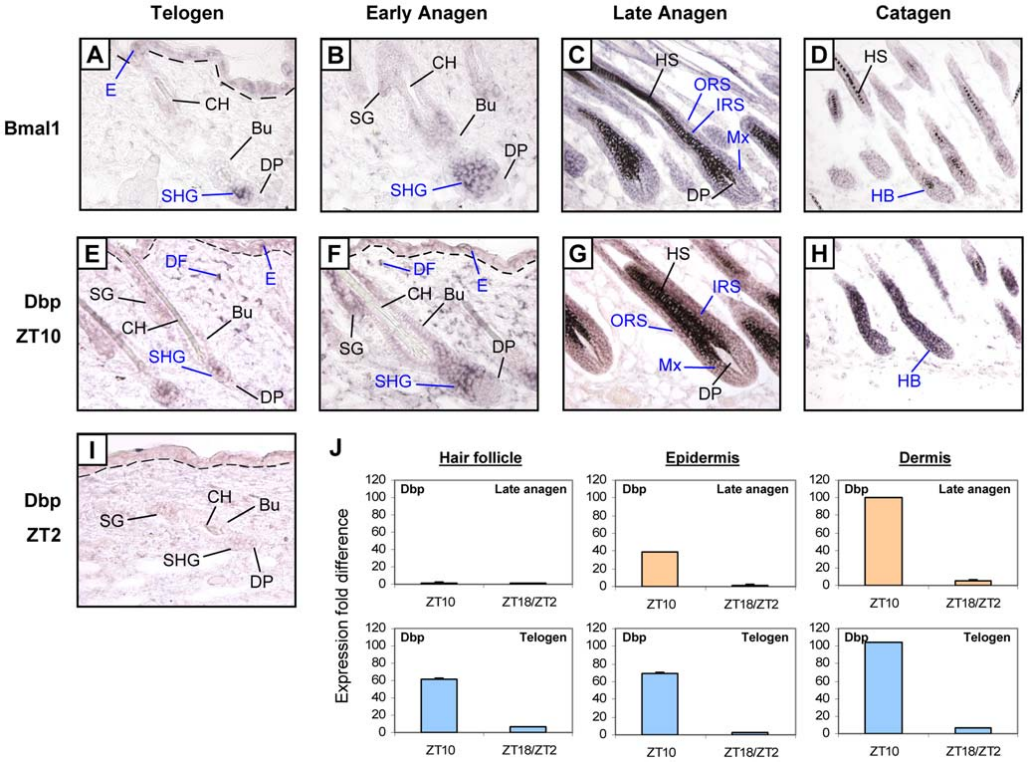
*Bmal1* and its target gene *Dbp* are co-expressed during hair follicle cycling. *In situ* hybridization staining of telogen, early anagen, late anagen, and catagen dorsal skin at ZT10 with *Bmal1* (A–D) and *Dbp* (E–H) probes. (I) *Dbp* expression at ZT2 in telogen skin. Dashed lines indicate border between epidermis and dermis. Note that the black pigment of the hair shaft in late anagen hair follicles is not hybridization signal. Bu – bulge, CH – club hair, DP – dermal papilla, HB – hair bulb, HS – hair shaft, IRS – inner root sheath, Mx – matrix, ORS – outer root sheath, SHG – secondary hair germ, SG – sebaceous gland. (J) *Dbp* expression from laser capture microdissected hair follicles, dermis, and epidermis for telogen and late anagen dorsal skin at ZT10 and ZT18/ZT2. Standard deviations were determined by using three replicates normalized to *Gapdh*. Ct values indicate detectable expression of clock genes in every sample, and fold was calculated relative to the lowest expression sample.

To identify the potential site of action for circadian mechanisms, we characterized the *in situ* skin expression of *Bmal1* and its target gene *Dbp*, a robust marker for clock output [18]. During telogen and early anagen, the most striking site of expression for both genes is in the secondary hair germ, an epithelial compartment located next to the dermal papilla (Figure 4 and Figure S3A). Interestingly, the secondary hair germ is derived from hair follicle bulge epithelial stem cells [29] and contains Lgr5-positive cells, a recently described multipotent stem cell population that is actively proliferating [30]. Keratinocytes of this compartment are the first to show robust proliferation at the onset of anagen [3], and are the progenitor cells for the anagen hair bulb, pigmented hair shaft, and inner root sheath [31]–[33]. As expected, *Dbp* expression is attenuated in the secondary hair germ of telogen hair follicles at ZT2 (Figure 4I). *Bmal1* and *Dbp* are also expressed in the lower regions of the late anagen and catagen hair follicles, as well as the epidermis and dermis throughout the hair growth cycle. Q-PCR of laser captured microdissected hair follicles, dermis, and epidermis indicate that clock genes are differentially expressed between ZT10 (high) and ZT18/ZT2 (low) in the dermis and epidermis during both telogen and late anagen (Figure 4J and Figure S4), consistent with previous studies in human skin [23],[25]. Interestingly, there is more active circadian regulation of these genes within the hair follicle proper during telogen compared to late anagen (Figure 4J and Figure S4), which likely contributes to the increased amplitude in expression during telogen. In summary, these data show that *Bmal1* and *Dbp* are widely expressed within hair follicles and other skin compartments throughout hair follicle cycling. However, during the important stage of initiation of hair growth at telogen and early anagen, the expression of these genes is prominent in the secondary hair germ of the hair follicle.

### Activation of CLOCK–Regulated Genes in Skin Is CLOCK/BMAL1–Dependent

**Figure 5:**
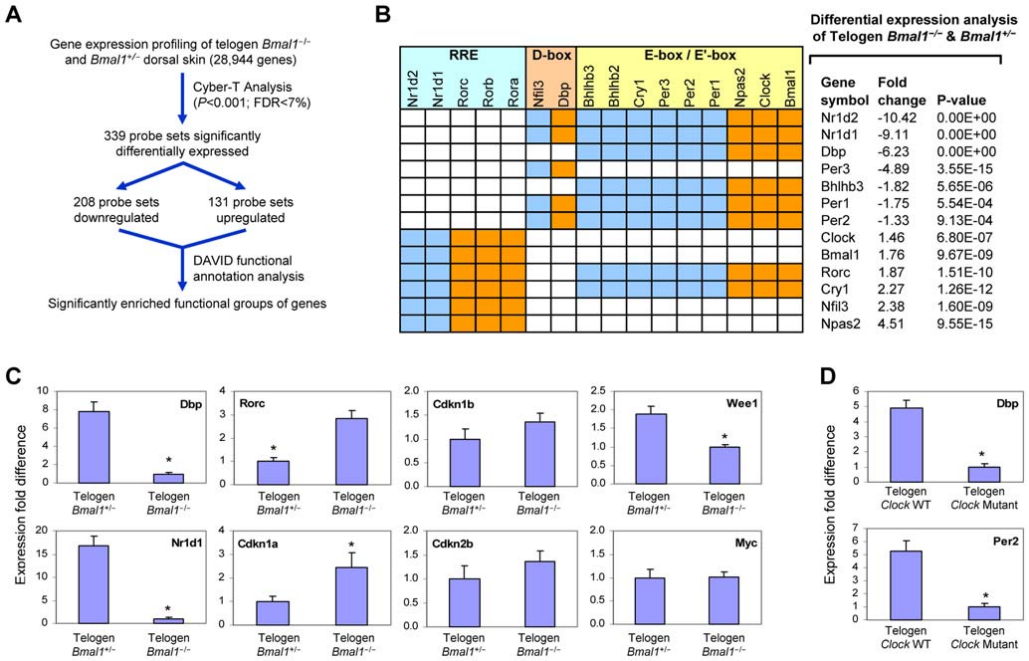
Significant differential expression of circadian transcriptional circuit and key cell cycle regulators in telogen *Bmal1* and *Clock* mutant dorsal skin. (A) Overview of microarray analysis of P22 *Bmal1* ^−/−^ and *Bmal1* ^+/−^ dorsal skin. FDR – false discovery rate. (B) The statistical differential expression of clock genes is shown with the circadian transcriptional circuit, which is represented as a matrix based on the results of Ueda et al. [17]. In the matrix, each column represents a gene encoding a transcription factor (grouped into three classes based on their binding sites: E-box/E′-box, D-box, and RRE), and each row represents a gene that is regulated by these transcription factors. Orange cells denote positive regulation and blue cells denote negative regulation. (C) Q-PCR of CLOCK-regulated genes and key cell cycle regulators in P22 *Bmal1* ^−/−^ and *Bmal1* ^+/−^ dorsal skin. (D) Q-PCR of CLOCK-regulated genes in P23 *Clock* wild-type and *Clock* mutant dorsal skin. For (C) and (D), expression is normalized to *Gapdh* and asterisks denote statistically significant (*P*<0.01) difference in expression between the littermates of the two genotypes. Error bars represent the S.E.M. for independent measurements from five *Bmal1* ^−/−^ and four *Bmal1* ^+/−^ mice, and three *Clock* wild-type and two *Clock* mutant mice.

To determine whether the expression of CLOCK-regulated genes in skin is dependent on CLOCK/BMAL1 activation, we profiled the dorsal skin of *Bmal1* ^−/−^ and *Bmal1* ^+/−^ control littermates at P22, when the hair follicles for both genotypes are in the first synchronized telogen (Figure 5A). Consistent with the mammalian circadian transcriptional circuit, functional annotation analysis of the 339 probe sets that are significantly differentially expressed in *Bmal1* ^−/−^ skin identified statistical enrichment (*P* = 5.7E-8) of genes annotated with the GO category of *rhythmic processes* (i.e., circadian clock genes). Specifically, clock genes with E/E′ or D-box in their regulatory regions (e.g., *Nr1d1*, *Nr1d2*, *Per1*, *Per2*, *Per3*, *Dbp*, and *Bhlhb3*) are significantly downregulated in *Bmal1* ^−/−^ mice (Figure 5B) [17], consistent with attenuated transcriptional activation by the CLOCK/BMAL1 heterodimer. The downregulation of *Nr1d1* and *Nr1d2* relieves the transcriptional repression of clock genes with RREs in their regulatory regions (e.g., *Clock*, *Bmal1*, *Npas2*, *Cry1*, *Nfil3*, and *Rorc*), resulting in significant upregulation of RRE-containing clock genes in *Bmal1* ^−/−^ skin (Figure 5B) [17]. We confirmed by Q-PCR the significant differential expression of CLOCK-regulated genes in *Bmal1* and *Clock* mutant skin (Figure 5C and 5D), indicating that the activation of these genes is CLOCK/BMAL1-dependent in skin.

A particularly intriguing finding of the microarray analysis of *Bmal1* ^−/−^ skin is the significant differential expression of *Rorc* and *Nr1d1* (Figure 5B). *Rorc*, which was recently shown to be a critical transcriptional activator of the G1 cell cycle regulator *Cdkn1a* (*p21*) [34], is upregulated approximately 3-fold (Figure 5C). Conversely, *Nr1d1*, a repressor of *Cdkn1a* expression [34], is downregulated approximately 15-fold. Consistent with these changes, we found that *Cdkn1a* is upregulated 2.5-fold (Figure 5C). With the exception of *Wee1*, which has been shown to be a CLOCK/BMAL1 target gene [35], other cell cycle regulators (e.g., *Cdkn1b*, *Cdkn2b* and *Myc*) are not significantly differentially expressed in *Bmal1* ^−/−^ skin (Figure 5C). Together, these results show that *Cdkn1a*, an important inhibitor of cell cycle progression, is upregulated in the skin of *Bmal1* ^−/−^ mice, suggesting the possibility that clock genes may regulate cell proliferation within skin.

### Delayed Anagen Progression in *Bmal1* and *Clock* Mutant Mice

**Figure 6:**
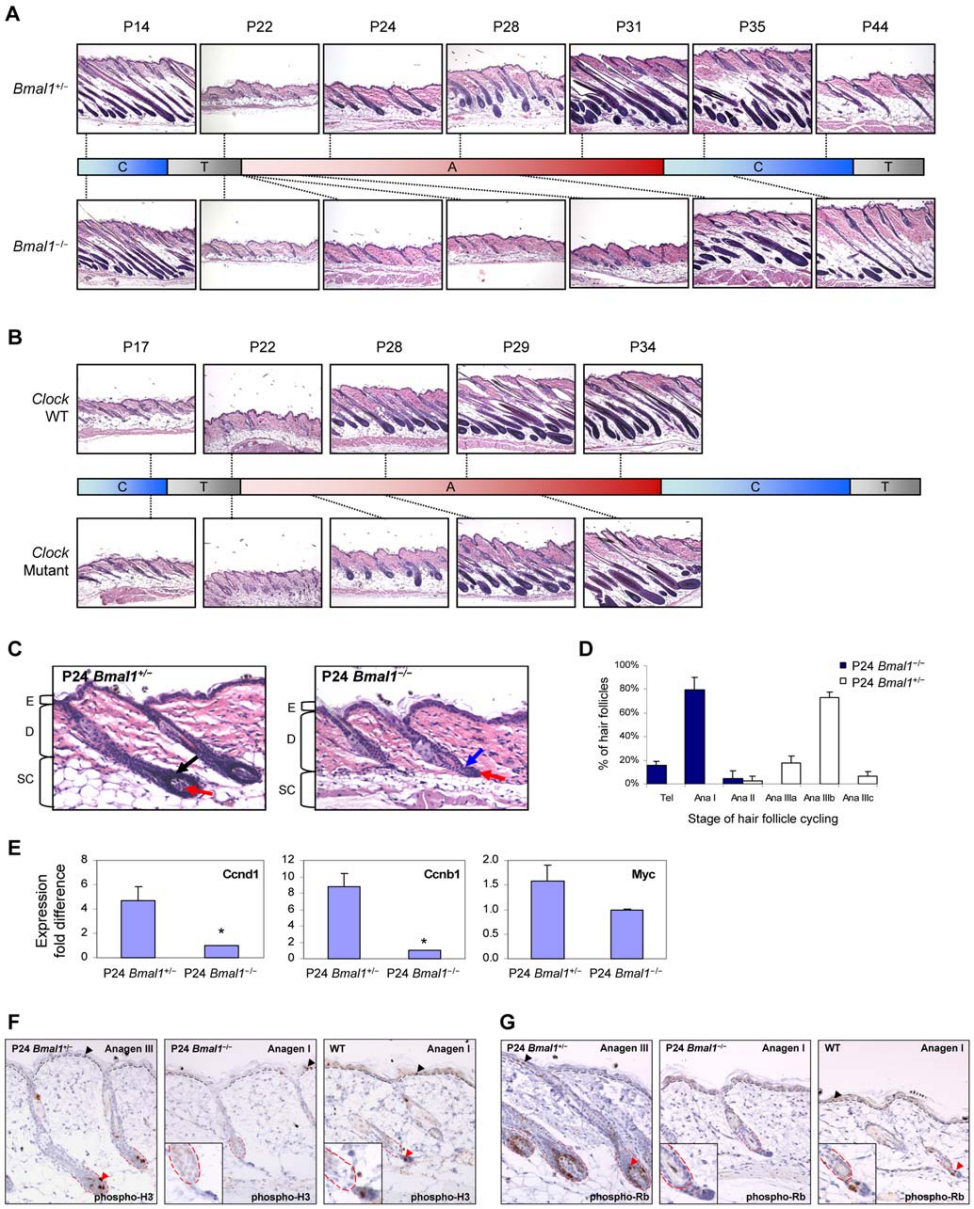
*Bmal1* and *Clock* regulate anagen progression in hair follicle cycling. (A) Representative histological sections of dorsal skin from *Bmal1* ^−/−^ mice and their gender-matched *Bmal1* ^+/−^ littermates at the indicated postnatal age (P). (B) Representative histological sections of dorsal skin from *Clock* mutant and their gender-matched wild-type (WT) littermates at the indicated postnatal age (P). For (A) and (B), time points are mapped (indicated by dotted line) based on histology to the corresponding phases of the hair growth cycle: anagen (A), catagen (C), and telogen (T). (C) Delayed anagen progression in *Bmal1* ^−/−^ mice compared to normal progression in *Bmal1* ^+/−^ littermates. At postnatal day 24, hair follicles are in anagen IIIb for the shown *Bmal1* ^+/−^ dorsal skin section; matrix cells (black arrow) form the enlarged hair bulb and the dermal papilla (red arrow) is larger than a third of bulb diameter. Note that the bulb is located in the middle of the subcutaneous adipose layer. Hair follicles are in anagen I for the shown *Bmal1* ^−/−^ dorsal skin section; thickening of keratinocyte strand (blue arrow) between the dermal papilla (red arrow) and the club hair. Note that the bulb is located in the dermis. Brackets indicate the different layers of the skin: E – epidermis, D – dermis, SC – subcutaneous adipose layer. (D) Quantitative hair cycle histomorphometric analysis. Percentage of hair follicles at the indicated hair cycle stage is based on staging fifty unique hair follicles for each genotype from three *Bmal1* ^+/−^ and two *Bmal1* ^−/−^ littermates at P24. Tel – telogen. Ana – anagen (Roman numerals indicate specific stages within anagen). (E) Q-PCR of *Ccnd1*, *Ccnb1*, and *Myc* in P24 *Bmal1* ^−/−^ and *Bmal1* ^+/−^ dorsal skin. Expression is normalized to *Gapdh* and error bars represent the S.E.M. for independent measurements from six *Bmal1* ^+/−^ and two *Bmal1* ^−/−^ littermates. Asterisks denote statistically significant (*P*<0.01) difference in expression between the two genotypes. Immunostaining of dorsal skin from P24 *Bmal1* ^+/−^ (left panel), *Bmal1* ^−/−^ (central panel) littermates using anti-phospho-histone H3 (F) and anti-phospho-Rb (Ser807/811) (G). The right panels of A and B are wild-type mice at P23 with hair follicles at equivalent stage of the hair growth cycle (anagen I) to the P24 *Bmal1* ^−/−^ mice. The insets are higher magnification of the lower regions of hair follicles. Black arrowheads; cells stained positive in the epidermis. Black dashed line; border between epidermis and dermis. Red dashed line; hair follicle bulb. Red arrowhead; cells stained positive within the hair follicle.

To determine whether circadian regulators play a functional role in the hair growth cycle, we studied the progression of synchronized hair follicle cycling in *Bmal1* ^−/−^ mice [36]. While the completion of hair follicle morphogenesis (P14) and entry into telogen (P21) are normal, there is a clear delay at the first synchronized anagen in *Bmal1* ^−/−^ mice (Figure 6A and Table S3). At P24 and P28, all *Bmal1* ^+/−^ mice have entered mid anagen, while their knockout littermates are still in the first stage of anagen; hair follicles of *Bmal1* ^−/−^ mice contain a thickened keratinocyte strand between the dermal papilla and the club hair but lack the highly proliferative matrix required for downward growth of the hair follicle during anagen (Figure 6C). The delay in anagen progression is confirmed by detailed quantitative hair cycle histomorphometric analysis and downregulation of *Myc*, as well as *cyclins D1* and *B1* (Figure 6D and 6E), which are normally upregulated by mid-anagen. By the time *Bmal1* ^−/−^ mice progress past the initial stage of anagen at P31, *Bmal1* ^+/−^ mice are already well advanced into late anagen (Figure 6A). When *Bmal1* ^+/−^ mice are in late catagen at P44, their knockout littermates are just beginning to enter catagen; the delay in anagen progression results in an overall shift of the hair growth cycle, while the duration of the entire hair growth cycle is not altered in *Bmal1* ^−/−^ mice. In *Clock* mutant mice [37],[38], we found a similar but less prominent delay in progression into the first synchronized anagen (Figure 6B and Table S4). We observed no obvious morphological abnormalities in the hair follicles of *Bmal1* and *Clock* mutant mice (Figure S5A). Hence, the delayed anagen progression in both *Clock* and *Bmal1* mutant mice—without abnormalities in hair follicle morphogenesis, basic architecture, pigmentation, and hair shaft formation—points to clock genes as important regulators of timing in hair follicle cycling.

### Circadian Clock Genes Regulate Cell Cycle Progression in the Secondary Hair Germ of Hair Follicles

Since circadian mechanisms are implicated in cell cycle control [34],[35],[39],[40], and we found upregulation of *Cdkn1a* in *Bmal1* ^−/−^ mouse skin, we next examined whether *Bmal1* ^−/−^ hair follicles exhibit alterations in cell cycle control. Interestingly, we observed an absence of mitotic cells within the hair follicles delayed at anagen I of P24 *Bmal1* ^−/−^ mice (Figure 6F); both *Bmal1* ^+/−^ and *Bmal1* ^−/−^ littermates have mitotic cells interspersed in the basal layer of epidermis, indicating a hair follicle-specific effect of the *Bmal1* mutation. This is significant because hair follicles of P23 wild-type mice, which are morphologically equivalent (in anagen I) to hair follicles of P24 *Bmal1* ^−/−^ mice, are molecularly distinct in that they contain mitotic cells in the secondary hair germ (Figure 6F, third panel), the site of high *Dbp* expression during early anagen. Both highly proliferative matrix cells of P24 *Bmal1* ^+/−^ hair follicles and the secondary hair germ cells of P23 wild-type hair follicles contain phosphorylated Rb (Figure 6G). In contrast, phosphorylated Rb was not detected in the secondary hair germ cells of P24 *Bmal1* ^−/−^ hair follicles of the same hair cycle stage (Figure 6G, third panel), indicating a blockade of secondary hair germ cells in G1. Thus, circadian clock genes may control hair follicle cycling at least in part by regulating proliferation in the secondary hair germ of the early anagen follicle.

## Discussion

In summary, our findings demonstrate a novel role for circadian clock genes in modulating the progression of the hair growth cycle, a cyclic biological process that has a much longer duration than the diurnal period. The fact that hair follicles of *Bmal1* and *Clock* mutant mice are specifically impeded in the first stage of anagen, and that clock genes are highly expressed in the secondary hair germ during telogen and early anagen, points to an important role for circadian clock genes in the control of this stage of hair follicle cycling. The delay in anagen progression is likely due to a block at the G1 phase of the cell cycle in the secondary hair germ cells, as these cells lack phosphorylated Rb and *Cdkn1a* (*p21*) is upregulated in *Bmal1* ^−/−^ mice. Together, these results suggest that the circadian clock modulates progression of hair follicle cycling via its effect on the cell cycle. The recurrent cycling of hair follicles through growth, regression, rest, and then growth again is a classical example of a regenerating system [1]. Thus, it is interesting that clock mechanisms in proliferation seems to become especially important during tissue regeneration, as evident by the impaired liver regeneration after hepatectomy in *Cry*-deficient mice [35].

Anagen eventually proceeds in *Bmal1* and *Clock* mutant mice, and this could be due to the proposed modulatory role (i.e., non-obligatory) of the circadian clock in regulating the cell cycle machinery [12],[40]. A delay rather than a permanent block in anagen progression is also found in many of the previously described mutant mouse models of key regulators in hair follicle cycling [41],[42], which may be due to functional redundancies of bHLH PAS transcription factors. One of these transcription factors, NPAS2, has been shown to form heterodimers with BMAL1 to activate circadian-controlled genes in both central and peripheral tissues [43],[44]. Intriguingly, *Npas2* is regulated during hair follicle cycling (Table S1) and is significantly upregulated in *Bmal1* ^−/−^ skin (Figure 6C). A likely explanation for the less pronounced delay in anagen progression in *Clock* mutant compared to *Bmal1* ^−/−^ mice is the difference in the nature of the genetic defects (reduced transcriptional activity in *Clock* mutant and null-mutation in *Bmal1* ^−/−^ mice); similar differences were found in previous studies showing less severe circadian phenotypes in *Clock* mutant mice [12]. It is important to note that the observed anagen progression delay in *Bmal1* and *Clock* mutant mice is in the context of completely normal morphological features for all compartments of the skin, including hair follicles, sebaceous glands, epidermis, dermis, and subcutaneous adipose layer. The young *Bmal1* ^−/−^ mice (3 to 4 week-old mice) examined in this study do not exhibit the series of age-related pathologies previously found in older *Bmal1* ^−/−^ mice, such as decreased hair growth after shaving in 40-week-old mice with reduced subcutaneous adipose tissue [45]. We found no difference in the thickness of the subcutaneous adipose layer of *Bmal1* ^−/−^ and control dorsal skin at comparable stages of hair follicle morphogenesis and cycling (Figure S5B). Thus, the absence of morphological and systemic abnormalities indicates a specific effect of circadian clock genes on the timing of anagen progression.

A recent study by Tanioka et al. demonstrated that the skin has an intrinsic oscillating circadian clock similar to other peripheral tissues such as the liver [26]. Furthermore, their experiments with SCN-ablated mice point to the importance of the central clock in maintaining expression of the epidermal circadian clock [26]. This is consistent with a number of studies demonstrating a role of the central clock in coordinating other peripheral clocks [10]. Therefore, it is quite likely that the modulation of the hair growth cycle by clock genes could involve both central and peripheral mechanisms. In support of the importance of peripheral clock mechanisms, we found prominent upregulation of clock genes during early anagen in a specific location within the hair follicle, the secondary hair germ. Proliferation of keratinocytes of this compartment, which contains precursor cells to the hair follicle and possibly stem cells [30],[32], is activated at the start of anagen. In addition, keratinocytes of the secondary hair germ lack mitotic cells and phosphorylated Rb in *Bmal1* ^−/−^ hair follicles that are halted at early anagen. Thus, the correspondence between the temporal and spatial expression of clock genes on the one hand and the location of the cell proliferation defect within the hair follicle on the other hand suggests a contribution by peripheral mechanisms. Future studies will dissect further the relative importance of the SCN and peripheral clocks in modulation of the hair growth cycle.

Changes in the stability of clock proteins can offset mRNA changes, and this can potentially explain the relatively constant PER2 protein levels between telogen and late anagen dorsal skin (Figure S3C). Another possible explanation is that the mRNAs of core clock genes (e.g. PER2) are fluctuating less dramatically between the different hair cycle phases in comparison to clock output genes (e.g. DBP, NR1D1) (Figure 3). However, without a suitable DBP antibody for western/immunohistochemistry, we were unable to test whether the protein level of DBP changes similarly to the transcript during hair follicle cycling. In future investigations, we plan to study the levels and localization of circadian clock proteins throughout the hair growth cycle.

The initial discovery of circadian clock gene expression altering in phase with the hair growth cycle was based on the development of probabilistic models for detecting periodic gene expression from time-course profiling over multiple cycles. An important feature of our probabilistic models is the ability to take into account of gene expression changes to due to the initial hair follicle morphogenesis and injury response following hair depilation. In addition, our clustering of periodically expressed transcriptional regulators during the hair growth cycle provide a genome-wide landscape of transcriptional regulation of hair follicle cycling, which identifies both known and possible novel regulators. Hence, our computational approach can be applied to identify periodically expressed regulators for other cyclic biological systems.

In conclusion, our findings support the idea that classical circadian genes may be utilized to modulate the progression of non-circadian cyclic processes, such as the hair growth cycle, via cell cycle control. Since many fur-bearing mammals undergo seasonal molting, we speculate that circadian control mechanisms for hair follicles may have evolved to allow seasonal regulation of hair growth. Circadian control mechanisms have previously been suggested for seasonal breeding and reproductive cycling [46],[47], and it is likely that other cyclic biological processes on different time scales are modulated by the circadian clock genes.

## Materials and Methods

### Ethics Statement

All animals were handled in strict accordance with good animal practice as defined by the relevant national and/or local animal welfare bodies, and all animal work was approved by the appropriate committee.

### RNA Extraction and Time-Course Microarray Experiments

For profiling of second synchronized and depilation-induced hair growth cycle, the same upper-mid region of dorsal skin was excised from C57BL/6 mice at representative postnatal days. Depilation-induced hair growth cycle was performed by applying wax/rosin mixture on the dorsal skin of seven-week old mice (all follicles in telogen). Animals were maintained under alternating 12 hours light/dark cycles with lights on at 6AM, and dorsal skin tissues were collected within 4-hour window (noon to 4PM). Histological sections were used to classify each sample into specific phases/stages of the hair growth cycle based on established morphological guidelines [3]. Total RNA was isolated from adjacent dorsal skin using the TRIzol method (Invitrogen) and cleaned using RNeasy Mini Kit (Qiagen). Quality of cleaned-up RNA was assessed using the Agilent 2100 Bioanalyzer (Agilent) prior to hybridization to Affymetrix Mouse Genome 430 2.0 arrays as described [27]. For each time point, multiple biological replicates were profiled, with each sample separately hybridized to an array (a total of 69 arrays used for profiling three hair growth cycles). The entire time-course wild-type microarray datasets are deposited in the NCBI Gene Expression Omnibus (www.ncbi.nlm.nih.gov/geo, GSE11186).

### Preprocessing of Microarray Datasets

Based on Principle Component Analysis of all samples and expression of housekeeping genes (e.g., *beta-Actin*, *Gapdh*), sample outliers are removed from downstream analyses. To remove genes not expressed during the hair growth cycle, we used MAS 5.0 generated present/absent calls to filter out genes that are not expressed in the dorsal skin at any sampled time point. A two-component noise model (TCM) was then applied to transform the MAS 5.0 expression data to ensure uniform replicate variance across the range of expression intensities [27].

### Probabilistic Model for Identification of Periodic Expression Changes

To identify genes with periodic expression patterns across multiple hair growth cycles, we construct a two-component probabilistic model with periodic and background components. The model includes binary indicators of periodicity that select one of the two mixture components for each of the probe sets. We use Bayesian inference techniques to estimate whether the observed data for each of the probe sets is more consistent with periodic or background expression, resulting in the final ranking of probe set with respect to the posterior probability of periodicity (see Text S1). Periodic component of the model is shown in Figure 1C using the framework of directed graphical models and plate notation [48]. Nodes within the plate (outermost rectangle labeled “n”) correspond to a single probe set. The plate indicates that these nodes are repeated for each of the probe sets and they share a dependence on a set of common periodic profiles and other parameters Φ. Circular nodes are continuous variables; square nodes are binary indicators. Large shaded rectangles visually group variables for each of the individual cycles. For each probe set, the hypothetical ideal cycle profile (light blue nodes) is chosen from a mixture of possible profiles. Individual cycles (blue nodes) follow the corresponding time points of the ideal cycle, but the model allows for three types of systematic differences between the cycles. First, expression measured on different generations of the Affymetrix platform may differ by some probe set-specific additive offset (light green node). Second, involvement in the initial hair follicle morphogenesis for some probe sets (light orange node) may cause altered expression during the first two time points of the first cycle. Third, involvement of some other probe sets in injury response to depilation (another light orange node) may cause altered expression during the first two time points of the third cycle. Actual replicates (red nodes) are conditionally independent observations of the individual cycles with probe set-specific replicate variance (green nodes). Finally, only the red nodes in the model are observed. Parameters of the priors Φ are estimated from the data using empirical Bayes framework. Bayesian inference techniques are used to marginalize over all other latent nodes in order to infer the posterior probability of periodic expression for each of the probe sets, conditioned on the observed replicate data and the prior parameters.

### Determination of Gene Expression Changes Due to Tissue Composition Changes

Previous studies have shown that the thickness of different layers of skin (epidermis, dermis, subcutaneous adipose tissue, and hair follicle depth) is significantly altered during the hair growth cycle [3],[49]. Genes whose expression in skin changes simply due to differences in the tissue composition of skin during the hair growth cycle are less likely to play regulatory roles than genes that are regulated within cells. Hence, we used a computational approach to systematically distinguish these two types of differential expression. To identify gene expression changes that are solely caused by tissue composition change over the hair growth cycle, we built a two-component Gaussian mixture model, where one component captures the expression profiles for a set of marker genes that are due to tissue composition change and the other component captures the background. The profiles for the following marker genes of different cell types were used: *filaggrin* (probe set ID 1427268_at) and *loricrin* (1420183_at) for the cornified cells; *keratin 1* (1422481_at) and *keratin 10* (1452166_at) for the suprabasal cells; *vimentin* (1450641_at) and *S100a4* (1424542_at) for mesenchymal cells; *Mef2a* (1452347_at), *Mef2c* (1421027_a_at), and *desmin* (1426731_at) for myocytes. Their profiles all display a characteristic U-shaped pattern during the hair growth cycle, reflecting that these cell types make up a smaller percentage of total skin tissue during anagen compared to telogen. Other genes that are expressed in these cell types would display similar profiles to these marker genes, so the mixture model was applied to the 8433 periodically expressed probe sets. Using this mixture model, we found that 2040 probe sets have high posterior probabilities (>0.9) of periodic gene expression changes due to tissue composition change over the hair growth cycle. Excluding these probe sets results in 6393 probe sets that we define as hair cycle-regulated genes.

### Statistical Differential Expression Analysis

Hair cycle-regulated expression profiles of the second synchronized cycle were used for statistical differential expression analysis because it is not perturbed by processes such as morphogenesis and wound healing that occur during the first and depilation-induced hair growth cycles, respectively. The time points for second hair growth cycle are classified into different phases of the hair growth cycle based on established morphological guidelines [3] as follow: early anagen (P23, P25), mid anagen (P27), late anagen (P29), early catagen (P37, P39), mid catagen (P41), telogen (P44). One-way ANOVA was performed on the TCM-transformed values of samples from a particular phase of the cycle to samples from the previous phase to determine upregulated genes (*P*<0.01). Significant enrichment of GO Biological Process categories within the different sets of genes upregulated at specific phases of the cycle was determined using DAVID functional annotation analysis [50]. Transcriptional regulators were systematically identified from the list of hair cycle-regulated genes by searching for the annotation category “regulation of transcription” in the GO Biological Process annotations. Using Partek Genomics Suite, partitioning cluster analysis with Euclidean distance was performed to group the second hair growth cycle TCM-transformed profiles of the transcriptional regulators.

### Quantitative Real-Time PCR

For cDNA synthesis of RNA (1 mg as input) extracted from dorsal skin, High Capacity cDNA Reverse Transcription Kit (Applied Biosystems) was performed as described [27]. For cDNA synthesis of RNA isolated from LCM samples, Sensiscript RT Kit (Qiagen) was performed following manufacture's protocol. The following TaqMan Gene Expression Assays (Applied Biosystems) were used. For circadian-regulated genes: *Bmal1* (*Arntl*, Mm00500226_m1), *Clock* (Mm00455941_m1), *Cry2* (Mm00546062_m1), *Dbp* (Mm01194021_m1), *Nr1d1* (Mm00520708_m1), *Per2* (Mm01285621_m1), and *Rorc* (Mm01261019_g1). For cell cycle-related genes: *Ccnd1* (Mm00432359_m1), *Ccnb1* (Mm00838401_m1), *Cdkn1a* (Mm00432448_m1), *Cdkn1b* (Mm00438167_g1), *Cdkn2b* (Mm00483241_m1), *Myc* (Mm00487803_m1), and *Wee1* (Mm00494175_m1). Three measurement replicates was performed to determine the expression level (critical threshold value) per sample, and the expression for each sample is normalized to the endogenous control gene, *Gapdh* (Mm99999915_g1).

### Western Blotting

Whole cell lysates from telogen (P20) and late anagen (P30) mouse dorsal skin were collected at ZT16. The following primary antibodies were used for overnight incubation: rabbit anti-mouse PER2 (Affinity Bioreagents, 1∶500 dilution) and rabbit anti-mouse GAPDH (Ambion, 1∶1000 dilution). Secondary antibody used is peroxidase-conjugated affinity pure goat anti-rabbit polyclonal antibody (Jackson ImmunoResearch, 1∶10000 dilution).

### Circadian Experiments

Two groups of C57BL/6 mice (104 mice total) were used for circadian experiments: P30 (late anagen; anagen IV to VI) and P46 (telogen). These two time points were selected because the follicles stay in late anagen and telogen phases for several consecutive days (verified with histological sections). Prior and during the experiments, the mice were carefully housed under alternating 12 hours light/dark cycles. For each group, four mice were sacrificed every four hours over the course of 48 hours, and total RNA was extracted from the same upper-mid region of dorsal skin as well as the liver for control. Similar circadian *Dbp* expression levels were found for liver taken from P30 and P46 mice.

### Laser Capture Microdissection

The excised dorsal skin was immediately embedded in O.C.T. Compound (Sakura Finetek USA, Torrance, CA), frozen in dry ice and stored at −80°C. Serial cryostat sections (8 µm thickness) were cut and mounted on autoclaved polytarthalene (PET) foil stretched on a metal frame (Leica). Tissue was fixed in cold acetone for 2 min and stained using Arcturus HistoGene LCM Frozen Section Staining Kit (Arcturus, Mountain View, CA) according to manufacturer's protocol. PET foil metal frames were mounted on a Leica AS LMD system (Leica) with the section facing downwards. Laser and microscope settings were as follow: 150× objective, aperture 6, intensity 46. The pulsed UV laser beam was carefully directed along the borders of each of the structures: whole hair follicles, epidermis, and dermis. Cross-contamination is very minimal in LCM samples as verified by Q-PCR of the following specific markers: *loricrin* (epidermis), *vimentin* (high in dermis, low in hair follicle), and *keratin 14* (epidermis and hair follicle). The collection tube cap was filled with a guanidine isothiocyanate (GITC)-containing buffer (Buffer RLT, RNeasy Mini Kit, Qiagen, Hilden, Germany) for cell lysis and preservation of RNA integrity. Tissue collection was verified by inspecting the tube cap. Post collection microcentrifuge tubes were immediately vortexed for 1 min. Total RNA was isolated using the RNeasy Micro Kit (Qiagen) according to the manufacturer's recommendations, including the DNA digestion step, with an elution volume of 14 µl and the addition of poly-A carrier RNA to the lysate. Shown are the results from one of three independent LCM experiments that yielded similar quantitative measurements.

### *In Situ* Hybridization

Probes specific for *Bmal1* and *Dbp* was generated by PCR using total cDNA derived from C57BL/6 mouse dorsal skin. *Bmal1* forward (F-) and reverse (R-) primers: F-TTAGCCAATGTCCTGGAAGG R-GCGATGACCCTCTTATCCTG. *Dbp* forward (F-) and reverse (R-) primers: F-CCCACAGTTGCAAAGAGACA and R-ATATGTCAGTCACCCGCACA. The resulting PCR products were cloned into pSTP19 vector (Roche Applied Science) for generation of digoxigenin-labeled (Roche Applied Science) antisense and sense probes using SP6 and T7 RNA polymerases, respectively. The probe generation, hybridization, washing and signal detection (using NBT/BCIP alkaline phosphatase substrate solution) procedures were performed as described [51].

### *Clock* and *Bmal1* Mutant Mice

Total of 80 *Clock* mice and 93 *Bmal1* mice (both in C57BL/6J genetic background [36]–[38]) were genotyped and grouped by gender. Each mouse is classified into specific phases/stages of the hair growth cycle based on the majority of hair follicles using established morphological guidelines [3].

### Quantitative Hair Cycle Histomorphometric Analysis

Analysis was performed as previously described with minor modifications [52].

### Expression Profiling of Dorsal Skin from *Bmal1* ^−/−^ Mice

Histological sections were used to verify that the hair follicles are in the first synchronized telogen based on established morphological guidelines [3]. RNA extraction from dorsal skin, clean-up, and array hybridization were the same as that described for the time-course microarrays above. We profiled three *Bmal1* ^−/−^ and three *Bmal1* ^+/−^ littermates, with each sample separately hybridized to an array (Affymetrix Mouse Gene 1.0 ST arrays). Statistical analyses were performed using Cyber-T program [53]. The cutoff for significant differential expression is set at *P*-value of 0.001, which corresponds to a false discovery rate of within 7% based on the calculated posterior probability of differential expression. The wild-type and *Bmal1* mutant microarray datasets are deposited in the NCBI Gene Expression Omnibus (www.ncbi.nlm.nih.gov/geo, GSE14006).

### Immunohistochemistry

Dorsal skin was fixed in 10% formalin, paraffin-embedded and sectioned at 6-µm. Antigen-retrieval was performed by heating slides in 0.01 M citrate buffer (pH6) for 20 min using an autoclave oven. After quenching endogenous peroxidase activity with 3% hydrogen peroxide for 5 min, sections were permeabilized using 0.2% Triton-X for 5 min. We then applied DakoCytomation Protein Block Serum-Free (Dako) to the sections for 30 min. Next, the slides are incubated overnight at 4°C with the following primary antibodies: phospho-histone H3 (Upstate, 1∶1000), phospho-Rb Ser807/811 (Cell Signaling, 1∶100), Keratin 5 (Covance, 1∶1000), AE13 and AE15 (kindly provided by Dr. T. T. Sun, 1∶100). We applied 1∶500 biotinylated IgG secondary antibodies (Vector) for 1 hr, and then incubated in Vectastain elite ABC Reagent (Vector) for 30 min. The slides were stained using the DakoCytomation Liquid DAB+ Substrate Chromogen System (Dako), and counterstained using 1∶5 diluted hematoxylin followed by bluing reagent.

## Supporting Information

Figure S1 Exclusion of gene expression changes that correspond to tissue composition changes. (A) Expression profiles of marker genes that are altered as the tissue composition changes during the hair growth cycle. The marker genes for the different cell types are as follows: filaggrin and loricrin for the cornified cells, keratin 1 and 10 for the suprabasal cells, vimentin and S100a4 for mesenchymal cells, and Mef2a, 2c, and desmin for myocytes. (B) Mixture model identified genes that can be explained by tissue composition changes over the hair growth cycle. The x-axis is the posterior probability of gene expression changes due to tissue composition changes, and the y-axis is the number of probe sets within each range of posterior probabilities.
(0.07 MB PDF)

Figure S2 Time-course profiles of genes belonging to representative GO Biological Process categories found to be significantly enriched. The heat map was generated using profiling data from the second synchronized hair growth cycle. Expression levels are indicated by colorimetric ratio-scale. Time points are mapped based on histology to the corresponding phases of the hair growth cycle: anagen (A), catagen (C), and telogen (T).
(0.23 MB PDF)

Figure S3 Expression of circadian clock genes and proteins in mouse dorsal skin at different phases of the hair growth cycle. (A) *In situ* hybridization staining of telogen, early anagen, late anagen, and catagen dorsal skin at ZT10 with *Bmal1* (left column) and *Dbp* (right column) anti-sense probes. Note the black pigment of the hair shaft in late anagen hair follicles is not hybridization signal. (B) As negative control, *in situ* hybridization staining of telogen, early anagen, and late anagen dorsal skin at ZT10 with *Dbp* sense probes. Dashed lines indicate border between epidermis and dermis. Brackets indicate the different layers of the skin: E - epidermis, D - dermis, SC - subcutaneous adipose layer. Bu - bulge, CH - club hair, DP - dermal papilla, HB - hair bulb, HS - hair shaft, IRS - inner root sheath, Mx - matrix, ORS - outer root sheath, SHG - secondary hair germ, SG - sebaceous gland. (C) Levels of PER2 proteins are not significantly different between telogen (P20) and late anagen (P30). Shown are two independent whole cell lysates from mouse dorsal skin collected at ZT16.
(0.75 MB PDF)

Figure S4 Expression of circadian clock genes from laser capture microdissected skin compartments. Q-PCR of *Bmal1* (A), *Per2* (B) and *Cry2* (C) from LCM-hair follicles, dermis, and epidermis for telogen and late anagen dorsal skin at ZT10 and ZT18/ZT2. (D) Q-PCR of *Dbp* from laser capture microdissected hair follicles at telogen, early anagen (anagen I–II), mid anagen (anagen III), late anagen (anagen IV–VI). For all panels, standard deviations were determined by using three replicates normalized to *Gapdh*. Ct values indicate detectable expression of clock genes in every sample, and fold was calculated relative to the lowest expression sample.
(0.06 MB PDF)

Figure S5 No morphological abnormalities in skin and hair follicle in *Clock* and *Bmal1* mutant mice. (A) Hair follicle structures are normal in *Clock* and *Bmal1* mutant mice. The top row show the H&E sections of hair follicles in dorsal skin of *Clock* and *Bmal1* mutant mice and their control littermates at late anagen. The bottom three rows are the corresponding immunostainings of the following specific hair differentiation markers: AE13 (cortex and cuticle of the hair shaft), AE15 (inner root sheath and medulla of the hair shaft), K5 (outer root sheath). Note that this particular *Bmal1* ^−/−^ mouse has white fur coat and therefore the unpigmented hair shaft reveals expected AE15 staining in the medulla. (B) No difference in the thickness of the subcutaneous adipose layer of *Bmal1* ^−/−^ and control dorsal skin. Note we measured thickness for comparable stages of hair follicle cycling; *Bmal1* ^−/−^ mice reaches late anagen at P34-P35, and *Bmal1* ^+/+^ and *Bmal1* ^+/−^ mice reaches late anagen at P30-P31.
(0.80 MB PDF)

Table S1 Time-course profile clustering of hair-cycle regulated genes. Probe set ID corresponds to the array used for the second and depilation-induced hair growth cycles (Mouse Genome 430 2.0). Old probe set ID corresponds to the array used for the first hair growth cycle (Murine Genome U74Av2).Columns labeled 1–9 correspond to log-transformed, zero-mean gene expression profiles for the “ideal hair cycle.”
(2.04 MB XLS)

Table S2 List of genes previously reported to have hair cycle-dependent gene expression changes. Probe set ID corresponds to the array used for the second and depilation-induced hair growth cycles (Mouse Genome 430 2.0). Old probe set ID corresponds to the array used for the first hair growth cycle (Murine Genome U74Av2). pHC is the posterior probability of being periodically expressed during the hair growth cycle. Note some genes have multiple probe sets, which have different hybridization signals and thus result in differences in pHC values.
(0.08 MB PDF)

Table S3 Hair cycle staging of *Bmal1* knockout mice (−/−) and their control littermates (+/+ and +/−). For each postnatal day (P), mice are grouped by genotype and each mouse is classified into specific phases/stages of the hair growth cycle based on the majority of hair follicles using established morphological guidelines. In general, we noted a slightly more advanced hair cycle progression in male mice; the table includes both genders which accounts for most of the variation within each genotype, but does not explain the observed differences between genotypes. Hence, for hair cycling progression comparison across genotypes, we matched littermates by gender.
(0.06 MB PDF)

Table S4 Hair cycle staging of *Clock* mutant mice (*Cl/Cl*) and their control littermates (+/+ and *Cl*+). Methodology same as Table S3.
(0.06 MB PDF)

Text S1 Probabilistic model for detection of periodic profiles.
(4.01 MB PDF)
