# Supplementary figures and images for: Circadian Clock Genes Contribute to the Regulation of Hair Follicle Cycling
Source: PLoS Genet. 2009 Jul 24;5(7):e1000573. doi: 10.1371/journal.pgen.1000573 (PMC2705795; doi:10.1371/journal.pgen.1000573)

Figure S1

**A**

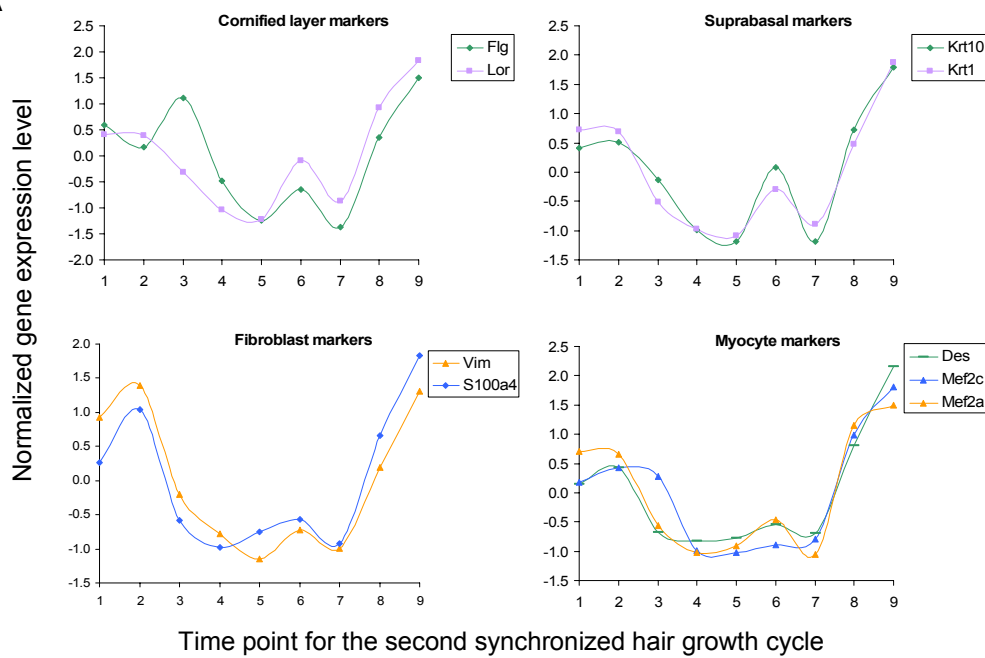

**B**

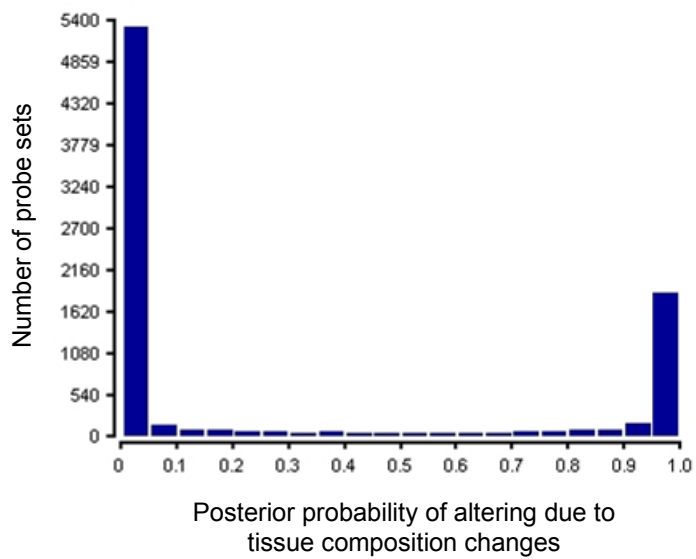

Supplement: Figure S1 — Exclusion of gene expression changes that correspond to tissue composition changes. (A) Expression profiles of marker genes that are altered as the tissue composition changes during the hair growth cycle. The marker genes for the different cell types are as follows: filaggrin and loricrin for the cornified cells, keratin 1 and 10 for the suprabasal cells, vimentin and S100a4 for mesenchymal cells, and Mef2a, 2c, and desmin for myocytes. (B) Mixture model identified genes that can be explained by tissue composition changes over the hair growth cycle. The x-axis is the posterior probability of gene expression changes due to tissue composition changes, and the y-axis is the number of probe sets within each range of posterior probabilities. (0.07 MB PDF) [file pgen.1000573.s001.pdf]

Figure S2

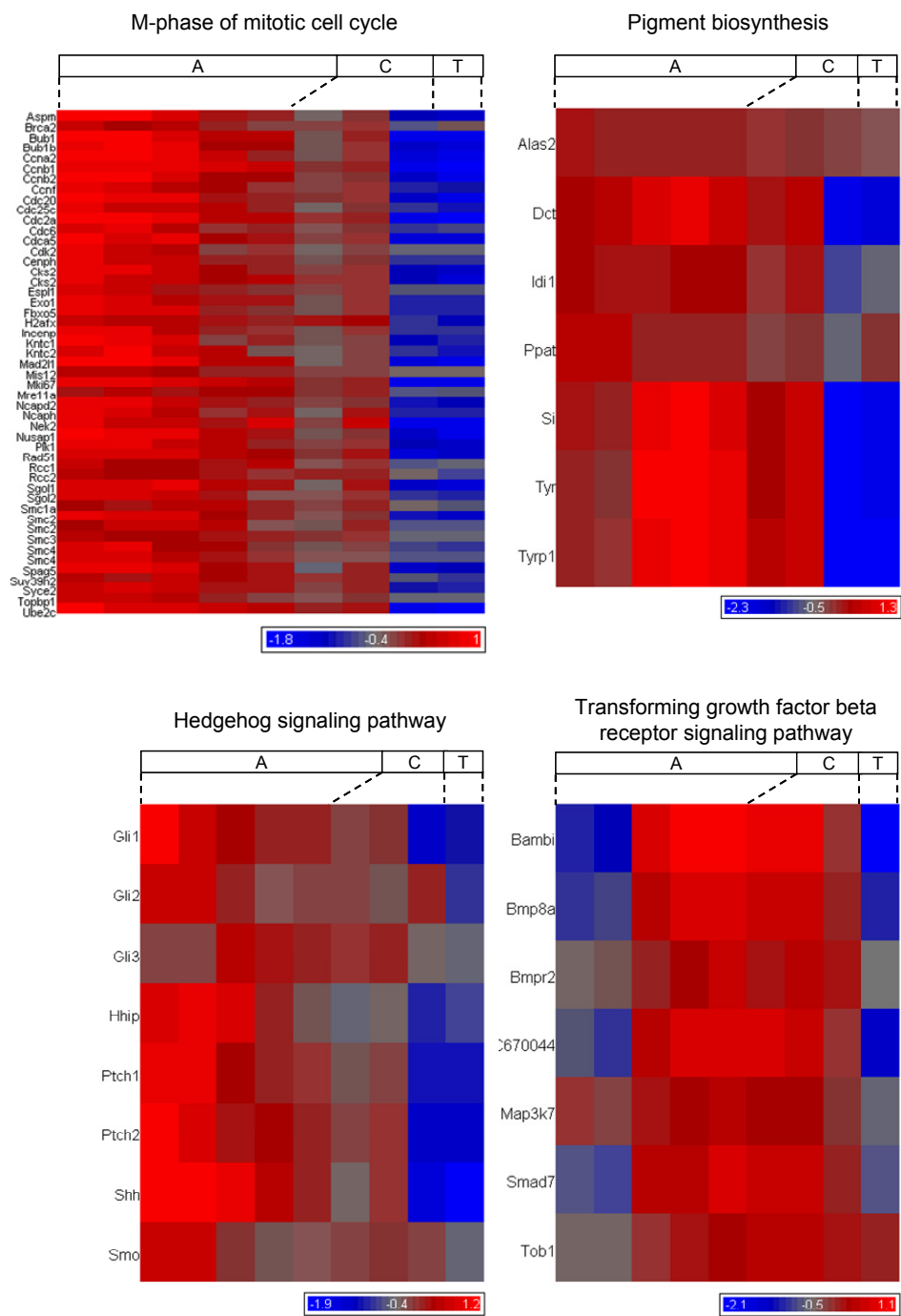

Supplement: Figure S2 — Time-course profiles of genes belonging to representative GO Biological Process categories found to be significantly enriched. The heat map was generated using profiling data from the second synchronized hair growth cycle. Expression levels are indicated by colorimetric ratio-scale. Time points are mapped based on histology to the corresponding phases of the hair growth cycle: anagen (A), catagen (C), and telogen (T). (0.23 MB PDF) [file pgen.1000573.s002.pdf]

**Figure S3**

**A**

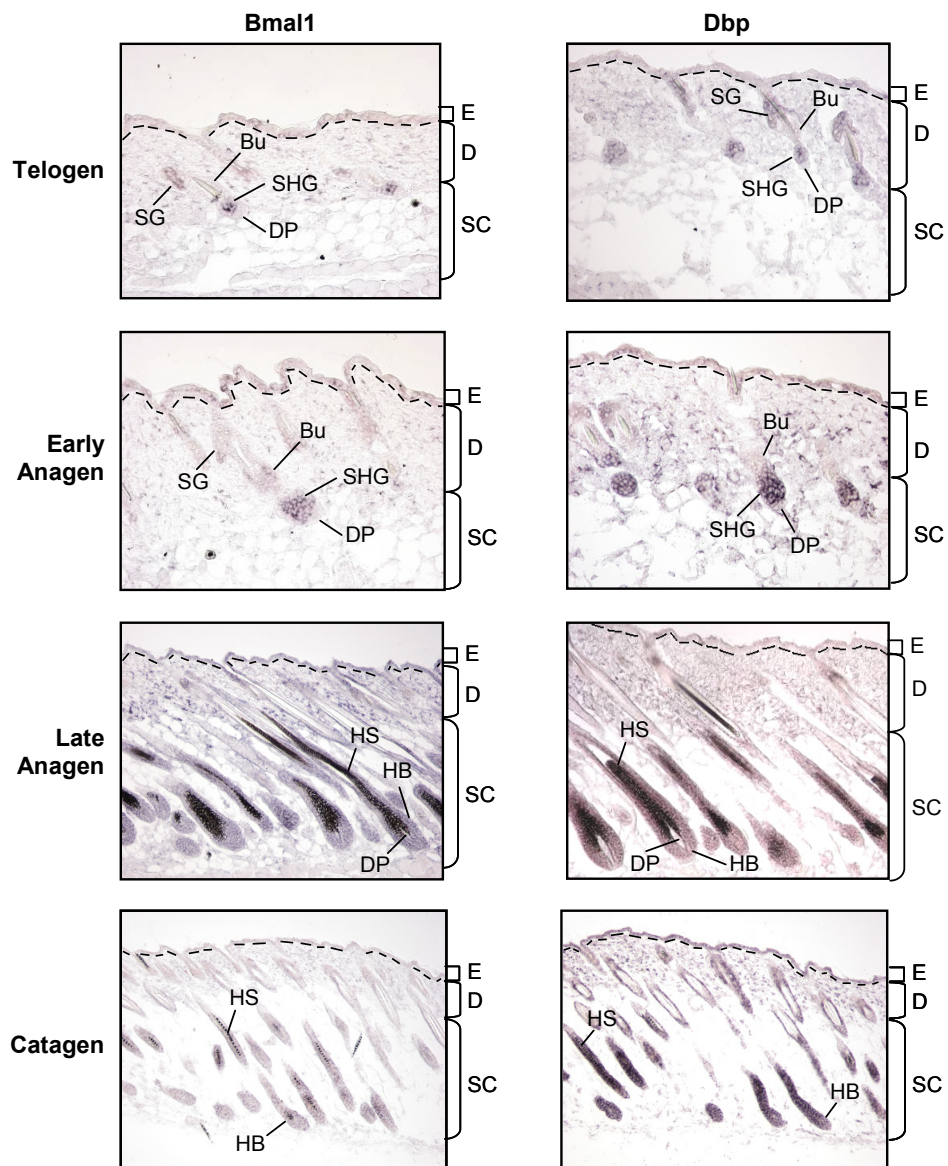

**B**

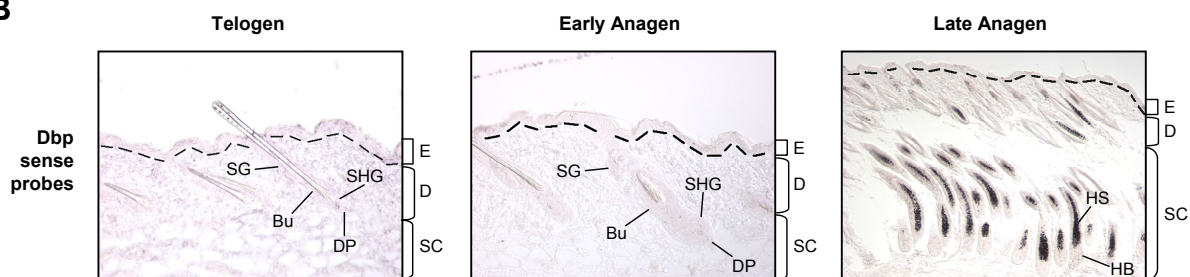

**C**

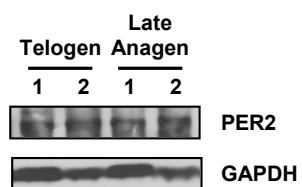

Supplement: Figure S3 — Expression of circadian clock genes and proteins in mouse dorsal skin at different phases of the hair growth cycle. (A) In situ hybridization staining of telogen, early anagen, late anagen, and catagen dorsal skin at ZT10 with Bmal1 (left column) and Dbp (right column) anti-sense probes. Note the black pigment of the hair shaft in late anagen hair follicles is not hybridization signal. (B) As negative control, in situ hybridization staining of telogen, early anagen, and late anagen dorsal skin at ZT10 with Dbp sense probes. Dashed lines indicate border between epidermis and dermis. Brackets indicate the different layers of the skin: E - epidermis, D - dermis, SC - subcutaneous adipose layer. Bu - bulge, CH - club hair, DP - dermal papilla, HB - hair bulb, HS - hair shaft, IRS - inner root sheath, Mx - matrix, ORS - outer root sheath, SHG - secondary hair germ, SG - sebaceous gland. (C) Levels of PER2 proteins are not significantly different between telogen (P20) and late anagen (P30). Shown are two independent whole cell lysates from mouse dorsal skin collected at ZT16. (0.75 MB PDF) [file pgen.1000573.s003.pdf]

Figure S4

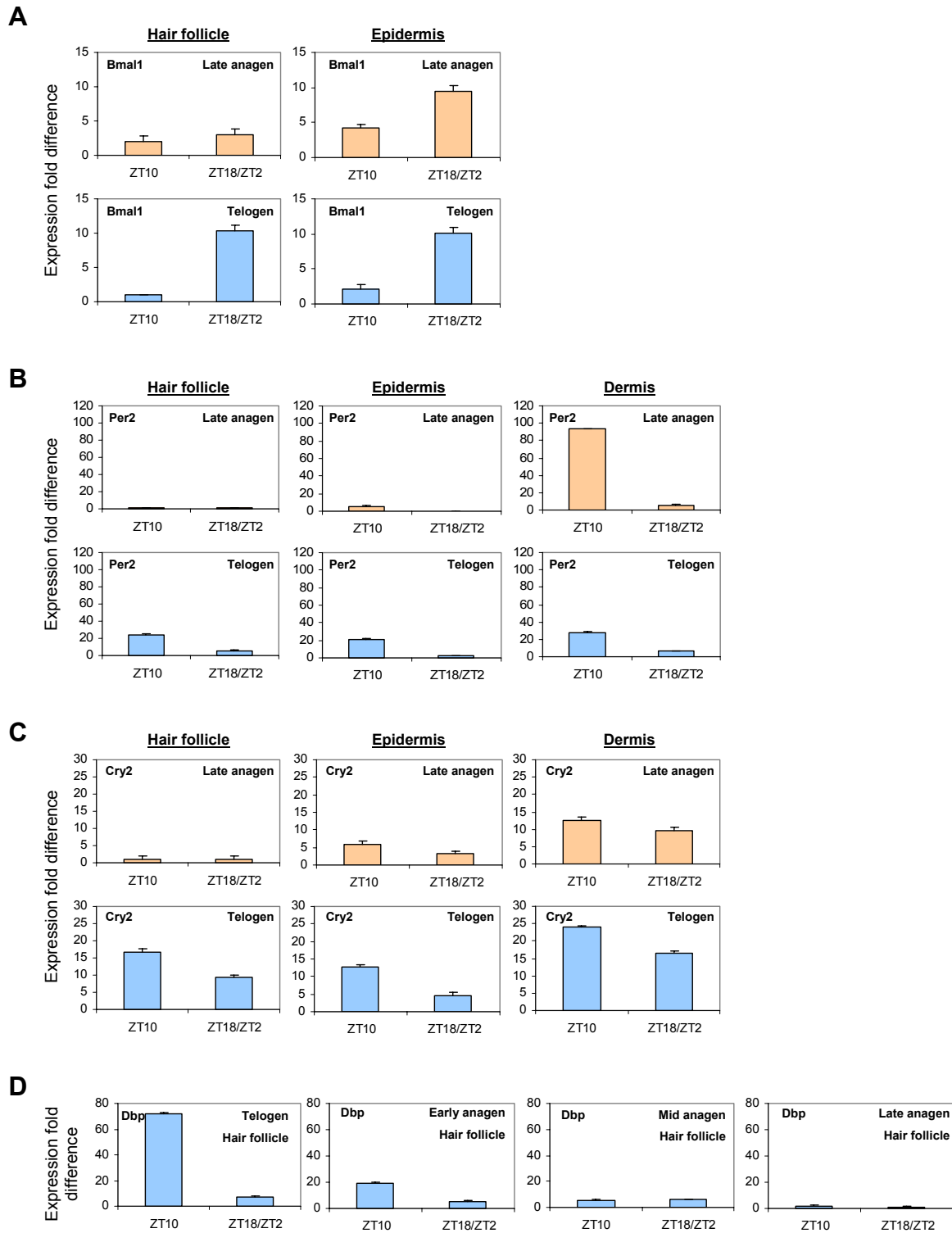

Supplement: Figure S4 — Expression of circadian clock genes from laser capture microdissected skin compartments. Q-PCR of Bmal1 (A), Per2 (B) and Cry2 (C) from LCM-hair follicles, dermis, and epidermis for telogen and late anagen dorsal skin at ZT10 and ZT18/ZT2. (D) Q-PCR of Dbp from laser capture microdissected hair follicles at telogen, early anagen (anagen I–II), mid anagen (anagen III), late anagen (anagen IV–VI). For all panels, standard deviations were determined by using three replicates normalized to Gapdh. Ct values indicate detectable expression of clock genes in every sample, and fold was calculated relative to the lowest expression sample. (0.06 MB PDF) [file pgen.1000573.s004.pdf]

**Figure S5**

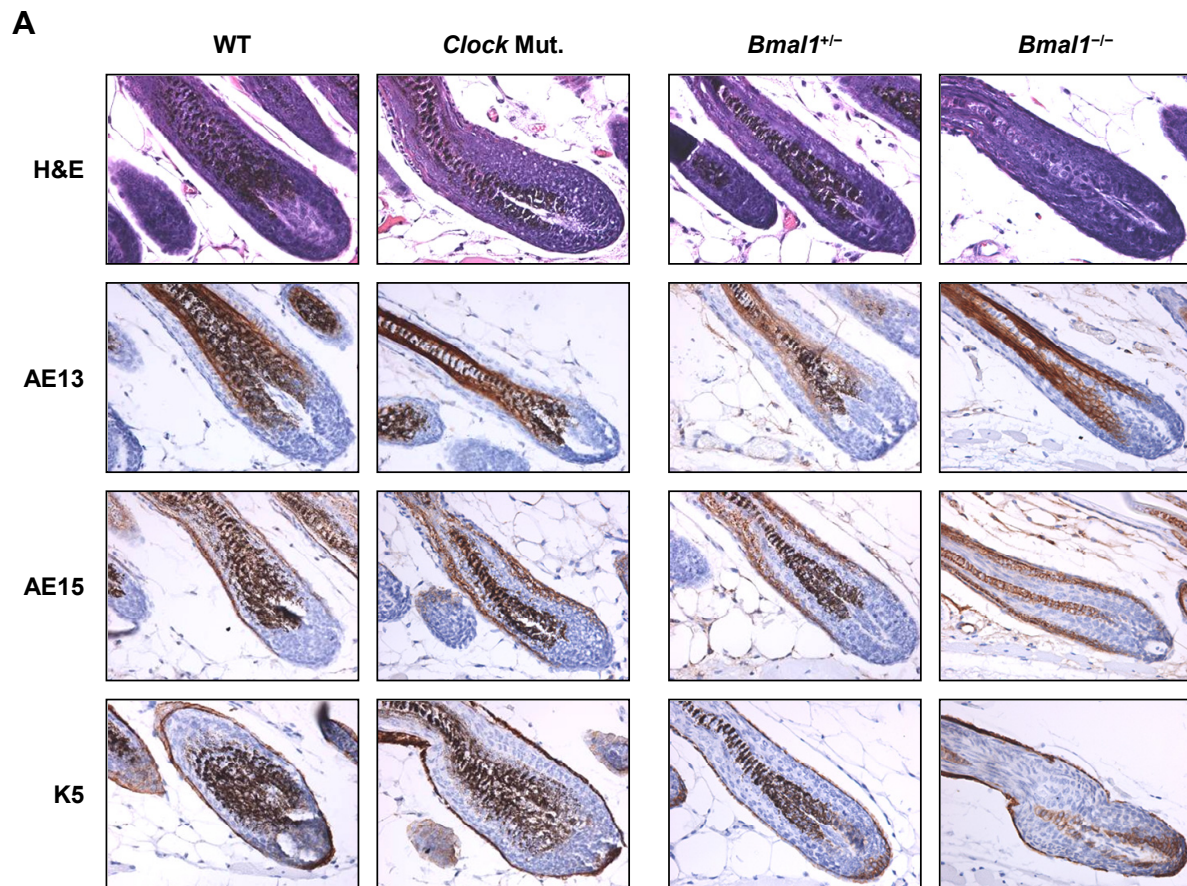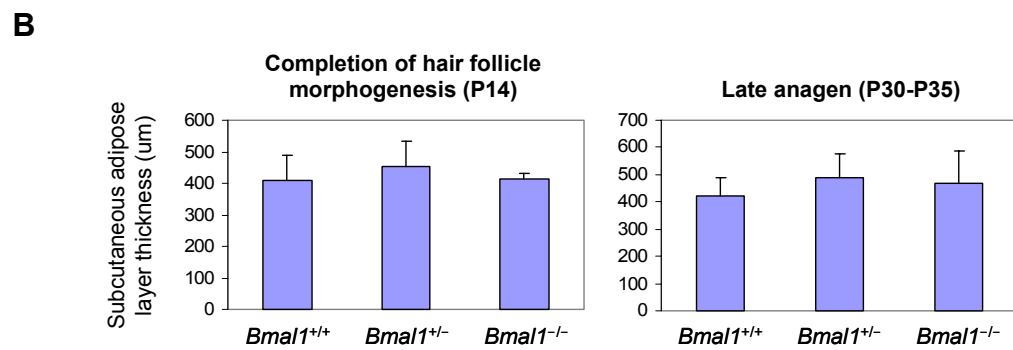

Supplement: Figure S5 — No morphological abnormalities in skin and hair follicle in Clock and Bmal1 mutant mice. (A) Hair follicle structures are normal in Clock and Bmal1 mutant mice. The top row show the H&E sections of hair follicles in dorsal skin of Clock and Bmal1 mutant mice and their control littermates at late anagen. The bottom three rows are the corresponding immunostainings of the following specific hair differentiation markers: AE13 (cortex and cuticle of the hair shaft), AE15 (inner root sheath and medulla of the hair shaft), K5 (outer root sheath). Note that this particular Bmal1 −/− mouse has white fur coat and therefore the unpigmented hair shaft reveals expected AE15 staining in the medulla. (B) No difference in the thickness of the subcutaneous adipose layer of Bmal1 −/− and control dorsal skin. Note we measured thickness for comparable stages of hair follicle cycling; Bmal1 −/− mice reaches late anagen at P34-P35, and Bmal1 +/+ and Bmal1 +/− mice reaches late anagen at P30-P31. (0.80 MB PDF) [file pgen.1000573.s005.pdf]
