# Supplementary material for: Circadian Clock Genes Contribute to the Regulation of Hair Follicle Cycling
Source: PLoS Genet. 2009 Jul 24;5(7):e1000573. doi: 10.1371/journal.pgen.1000573 (PMC2705795; doi:10.1371/journal.pgen.1000573)
Supplement: Table S2 — List of genes previously reported to have hair cycle-dependent gene expression changes. Probe set ID corresponds to the array used for the second and depilation-induced hair growth cycles (Mouse Genome 430 2.0). Old probe set ID corresponds to the array used for the first hair growth cycle (Murine Genome U74Av2). pHC is the posterior probability of being periodically expressed during the hair growth cycle. Note some genes have multiple probe sets, which have different hybridization signals and thus result in differences in pHC values. (0.08 MB PDF) [file pgen.1000573.s007.pdf]

Table S2

Probe Set	Old Probe Set	pHC	Gene Symbol	Gene Name	References
1458467_at		1	1110032D16Rik	RIKEN cDNA 1110032D16 gene	Ishimatsu-Tsuji Y, Moro O et al., JID 2005
1451382_at		1	1810008K03Rik	RIKEN cDNA 1810008K03 gene	Ishimatsu-Tsuji Y, Moro O et al., JID 2005
1430296_at		1	4733401H21Rik	RIKEN cDNA 4733401H21 gene	Ishimatsu-Tsuji Y, Moro O et al., JID 2005
1420415_at		1	AY026312	cDNA sequence AY026312	Ishimatsu-Tsuji Y, Moro O et al., JID 2005
1449970_at		1	Capn12	calpain 12	Dear TN, Meier NT et al. Genomics 2000
1421001_a_at	160647_at	1	Car6	carbonic anhydrase 6	Lin KK, Chudova D et al., PNAS 2004; Ishimatsu-Tsuj
1448261_at	98140_at	0	Cdh1	cadherin 1	Muller-Rover S, Tokura Y et al. Exp Dermatol 1999
1443509_at		0.53	Cdh1	Cadherin 1	---
1426673_at	100409_at	1	Cdh3	cadherin 3	Muller-Rover S, Tokura Y et al. Exp Dermatol 1999
1441255_at		0.03	Cdh3	Cadherin 3	---
1424638_at	94881_at	1	Cdkn1a	cyclin-dependent kinase inhibitor 1A (P21)	Mitsui S, Ohuchi A et al. J Derm Sci 2001
1421679_a_at	98067_at	1	Cdkn1a	cyclin-dependent kinase inhibitor 1A (P21)	---
1418626_a_at	95286_at	1	Clu	clusterin	Seiberg M, Marthinuss J. Dev Dyn 1995
1437458_x_at		1	Clu	clusterin	---
1437689_x_at		1	Clu	clusterin	---
1454849_x_at		1	Clu	clusterin	---
1416325_at	93122_at	1	Crisp1	cysteine-rich secretory protein 1	Lin KK, Chudova D et al., PNAS 2004
1416776_at	160937_at	1	Crym	crystallin, mu	Aoki N, Ito K et al. JID 2000
1416563_at	103341_at	1	Ctps	cytidine 5'-triphosphate synthase	Ishimatsu-Tsuji Y, Moro O et al., JID 2005
1418989_at	104696_at	1	Ctse	cathepsin E	Ishimatsu-Tsuji Y, Moro O et al., JID 2005
1427797_s_at		1	Ctse	cathepsin E	---
1423805_at	104633_at	1	Dab2	disabled homolog 2 (Drosophila)	Lin KK, Chudova D et al., PNAS 2004
1420498_a_at	98045_s_at	1	Dab2	disabled homolog 2 (Drosophila)	---
1429693_at		0.98	Dab2	disabled homolog 2 (Drosophila)	---
1430604_a_at		0	Dab2	disabled homolog 2 (Drosophila)	---
1415723_at	160265_at	0	Eif5	eukaryotic translation initiation factor 5	Ishimatsu-Tsuji Y, Moro O et al., JID 2005
1433631_at		0.98	Eif5	eukaryotic translation initiation factor 5	---
1454663_at		1	Eif5	eukaryotic translation initiation factor 5	---
1454664_a_at		1	Eif5	eukaryotic translation initiation factor 5	---
1456256_at		0.43	Eif5	eukaryotic translation initiation factor 5	---
1419555_at	103283_at	1	Elf5	E74-like factor 5	Lin KK, Chudova D et al., PNAS 2004
1419556_at		1	Elf5	E74-like factor 5	---
1435663_at		1	Esr1	estrogen receptor 1 (alpha)	Ohnemus U, Uenalan M et al. Endocrinology 2005
1457877_at		0.97	Esr1	Estrogen receptor 1 (alpha)	---
1460591_at		1	Esr1	estrogen receptor 1 (alpha)	---
1422540_at	94307_at	1	Fbln1	fibulin 1	Lin KK, Chudova D et al., PNAS 2004
1451119_a_at	94308_at	0.91	Fbln1	fibulin 1	---
1451119_a_at	94309_g_at	1	Fbln1	fibulin 1	---
1439688_at		0	Fbln1	fibulin 1	---
1423136_at		1	Fgf1	fibroblast growth factor 1	Kawano M, Komi-Kuramochi A et al. JID 2005
1450869_at		0.05	Fgf1	fibroblast growth factor 1	---
1418498_at	99893_at	0.63	Fgf13	fibroblast growth factor 13	Kawano M, Komi-Kuramochi A et al. JID 2005
1418497_at		1	Fgf13	fibroblast growth factor 13	---
1449545_at		1	Fgf18	fibroblast growth factor 18	Kawano M, Komi-Kuramochi A et al. JID 2005
1449826_a_at		0	Fgf2	fibroblast growth factor 2	Kawano M, Komi-Kuramochi A et al. JID 2005
1460296_a_at		1	Fgf22	fibroblast growth factor 22	Kawano M, Komi-Kuramochi A et al. JID 2005
1426186_a_at		1	Fgf5	fibroblast growth factor 5	Kawano M, Komi-Kuramochi A et al. JID 2005
1438883_at		1	Fgf5	fibroblast growth factor 5	---
1422243_at	99435_at	0.2	Fgf7	fibroblast growth factor 7	Kawano M, Komi-Kuramochi A et al. JID 2005
1438405_at		1	Fgf7	fibroblast growth factor 7	---
1424050_s_at	97509_f_at	1	Fgfr1	fibroblast growth factor receptor 1	Kawano M, Komi-Kuramochi A et al. JID 2005
1425911_a_at		1	Fgfr1	fibroblast growth factor receptor 1	---
1436551_at		1	Fgfr1	fibroblast growth factor receptor 1	---
1433489_s_at	93090_at	0.71	Fgfr2	fibroblast growth factor receptor 2	Kawano M, Komi-Kuramochi A et al. JID 2005
1420847_a_at	93091_s_at	0.08	Fgfr2	fibroblast growth factor receptor 2	---
1421841_at		1	Fgfr3	fibroblast growth factor receptor 3	Kawano M, Komi-Kuramochi A et al. JID 2005
1425796_a_at		1	Fgfr3	fibroblast growth factor receptor 3	---
1450508_at	92674_at	1	Foxn1	forkhead box N1	Meier N, Dear TN et al. Mech Dev 1999
1456815_at		1	Foxn1	Forkhead box N1	---
1450440_at	93872_at	0	Gfra1	glial cell line derived neurotrophic factor family	Botchkareva NV, Botchkarev VA et al. Am J Path 200
1439015_at		0.02	Gfra1	glial cell line derived neurotrophic factor family	---
1423007_a_at	92449_at	0.01	Gfra2	glial cell line derived neurotrophic factor family	Botchkareva NV, Botchkarev VA et al. Am J Path 200
1438650_x_at	100064_f_at	1	Gja1	gap junction membrane channel protein alpha	Risek B, Klier FG et al. Development 1992
1415800_at	100065_r_at	1	Gja1	gap junction membrane channel protein alpha	---
1415801_at		1	Gja1	gap junction membrane channel protein alpha	---
1437992_x_at		1	Gja1	gap junction membrane channel protein alpha	---
1438945_x_at		1	Gja1	gap junction membrane channel protein alpha	---
1438973_x_at		0.03	Gja1	gap junction membrane channel protein alpha	---
1423271_at	98423_at	1	Gjb2	gap junction membrane channel protein beta 2	Risek B, Klier FG et al. Development 1992
1423858_a_at		1	Hmgcs2	3-hydroxy-3-methylglutaryl-Coenzyme A synth	Schlake T, Beibel M et al. Gene Exp Patterns 2004
1431833_a_at		1	Hmgcs2	3-hydroxy-3-methylglutaryl-Coenzyme A synth	---
1424067_at	96752_at	1	Icam1	intercellular adhesion molecule	Muller-Rover S, Bulfone-Paus et al. J Histochem and
1421473_at	94755_at	0	Il1a	interleukin 1 alpha	Hoffmann R, Happle R et al. Eur J Derm 1998
1449399_a_at		0.02	Il1b	interleukin 1 beta	Hoffmann R, Happle R et al. Eur J Derm 1998
1448950_at	93914_at	0.01	Il1r1	interleukin 1 receptor, type I	Hoffmann R, Happle R et al. Eur J Derm 1998
1419532_at	102658_at	1	Il1r2	interleukin 1 receptor, type II	Hoffmann R, Happle R et al. Eur J Derm 1998
1457108_at		0.04	Il1r2	Interleukin 1 receptor, type II	---

Table S2 – cont.

Probe Set	Old Probe Set	pHC	Gene Symbol	Gene Name	References
1450678_at	102353_at	1	Itgb2	integrin beta 2	Muller-Rover S, Bulfone-Paus et al. J Histochem and
1415899_at	102362_i_at	1	Junb	Jun-B oncogene	Lin KK, Chudova D et al., PNAS 2004
1415899_at	102363_r_at	1	Junb	Jun-B oncogene	---
1452514_a_at	99956_at	1	Kit	kit oncogene	Peters EMJ, Maurer M et al. JID 2003
1415900_a_at		1	Kit	kit oncogene	---
1459588_at		0	Kit	Kit oncogene	---
1415855_at	99577_at	1	Kitl	kit ligand	Peters EMJ, Maurer M et al. JID 2003
1415854_at		0.12	Kitl	kit ligand	---
1426152_a_at		0.94	Kitl	kit ligand	---
1440621_at		0.72	Kitl	Kit ligand	---
1448117_at		0.73	Kitl	kit ligand	---
1427179_at	97814_at	1	Krt1-3	keratin complex 1, acidic, gene 3	Meier N, Dear TN et al. Mech Dev 1999
1448457_at	101569_at	1	Krt2-6g	keratin complex 2, basic, gene 6g	Porter Rm, Corden LD et al. British J Derm 2001
1452957_at	97469_at	1	Krtap3-3	keratin associated protein 3-3	Ishimatsu-Tsuji Y, Moro O et al., JID 2005
1450539_at	93710_at	1	Krtap5-1	keratin associated protein 5-1	Wood L, Mills M et al. JBC 1990
1450540_x_at		1	Krtap5-1	keratin associated protein 5-1	---
1422958_at	92240_at	1	Krtap5-4	keratin associated protein 5-4	Wood L, Mills M et al. JBC 1990
1451859_at	101374_at	1	Krtap6-1	keratin associated protein 6-1	Aoki N, Ito K et al. JBC 1997
1420751_at	101375_at	1	Krtap6-1	keratin associated protein 6-1	---
1425872_at	101376_at	1	Krtap6-1	keratin associated protein 6-1	---
1449919_at	92253_at	1	Krtap6-2	keratin associated protein 6-2	Aoki N, Ito K et al. JBC 1997
1427842_at	100735_at	1	Krtap6-3	keratin associated protein 6-3	Aoki N, Ito K et al. JBC 1997
1452575_at		1	Krtap6-3	keratin associated protein 6-3	---
1427211_at	102793_at	1	Krtap8-1	keratin associated protein 8-1	Aoki N, Ito K et al. JBC 1997
1421689_at	97152_at	1	Krtap8-2	keratin associated protein 8-2	Aoki N, Ito K et al. JBC 1997
1419707_at	92246_at	1	Krtap14	keratin associated protein 14	Aoki N, Ito K et al. JID 1998; Kuhn F, Lassing C et al.
1419507_at		1	Krtap15	keratin associated protein 15	Kuhn F, Lassing C et al. Mech Dev 1999
1422069_at	101161_at	1	Mc1r	melanocortin 1 receptor	Ermak G & Slominski A. JID 1997
1417234_at	100016_at	0.95	Mmp11	matrix metalloproteinase 11	Ishimatsu-Tsuji Y, Moro O et al., JID 2005
1419675_at		0.75	Ngfb	nerve growth factor, beta	Peters EM, Hendrix S et al. J Histochem Cytochem 2
1417155_at	103048_at	1	Nmyc1	neuroblastoma myc-related oncogene 1	Lin KK, Chudova D et al., PNAS 2004
1425922_a_at		1	Nmyc1	neuroblastoma myc-related oncogene 1	---
1434802_s_at		0.99	Ntf3	neurotrophin 3	Botchkarev VA, Welker P et al. Am J Path 1998
1450803_at		0.09	Ntf3	neurotrophin 3	---
1440353_at		0.37	Ntf5	neurotrophin 5	Botchkarev VA, Botchkareva NV et al. FASEB J 1999
1420838_at		0.02	Ntrk2	neurotrophic tyrosine kinase, receptor, type 2	Botchkarev VA, Botchkareva NV et al. FASEB J 1999
1435196_at		0.79	Ntrk2	neurotrophic tyrosine kinase, receptor, type 2	---
1422329_a_at		0	Ntrk3	neurotrophic tyrosine kinase, receptor, type 3	Botchkarev VA, Welker P et al. Am J Path 1998
1425071_s_at		0.03	Ntrk3	neurotrophic tyrosine kinase, receptor, type 3	---
1426003_at		0	Ntrk3	neurotrophic tyrosine kinase, receptor, type 3	---
1433825_at		0.87	Ntrk3	RIKEN cDNA E430016F16 gene	---
1417575_at		1	Otub2	OTU domain, ubiquitin aldehyde binding 2	Schlake T, Beibel M et al. Gene Exp Patterns 2004
1417576_a_at		1	Otub2	OTU domain, ubiquitin aldehyde binding 2	---
1418883_a_at	100720_at	1	Pabpc1	poly A binding protein, cytoplasmic 1	Ishimatsu-Tsuji Y, Moro O et al., JID 2005
1453840_at		0.99	Pabpc1	poly A binding protein, cytoplasmic 1	---
1427562_a_at		0.97	Prkca	protein kinase C, alpha	Li LF, Chang BD et al. J Dermatol 1999
1450945_at		1	Prkca	protein kinase C, alpha	---
1448556_at	95030_at	1	Prlr	prolactin receptor	Foitzik K, Krause K et al. Am J Pathology 2003
1425853_s_at		1	Prlr	prolactin receptor	---
1420467_at		1	Psors1c2	psoriasis susceptibility 1 candidate 2 (human)	Schlake T, Beibel M et al. Gene Exp Patterns 2004
1422324_a_at	104262_at	1	Pthlh	parathyroid hormone-like peptide	Cho YM, Woodard GL et al. JID 2003
1427527_a_at		1	Pthlh	parathyroid hormone-like peptide	---
1417092_at	98482_at	1	Pthr1	parathyroid hormone receptor 1	Cho YM, Woodard GL et al. JID 2003
1421856_at	101051_at	1	S100a3	S100 calcium binding protein A3	Kizawa K, Tsuchimoto S et al. JID 1998
1421856_at	101052_g_at	1	S100a3	S100 calcium binding protein A3	---
1421375_a_at	92770_at	1	S100a6	S100 calcium binding protein A6 (calcyclin)	Wood L, Carter D et al. JID 1991
1415964_at	94056_at	1	Scd1	stearoyl-Coenzyme A desaturase 1	Zheng Y, Eilertsen KJ et al. Nat Genet 1999
1415964_at	94057_g_at	1	Scd1	stearoyl-Coenzyme A desaturase 1	---
1415965_at		0.94	Scd1	stearoyl-Coenzyme A desaturase 1	---
1415822_at	95758_at	1	Scd2	stearoyl-Coenzyme A desaturase 2	Zheng Y, Eilertsen KJ et al. Nat Genet 1999
1415823_at		1	Scd2	stearoyl-Coenzyme A desaturase 2	---
1415824_at		1	Scd2	stearoyl-Coenzyme A desaturase 2	---
1423366_at		0.99	Scd3	stearoyl-coenzyme A desaturase 3	Zheng Y, Prouty SM et al. Genomics 2001
1450956_at		1	Scd3	stearoyl-coenzyme A desaturase 3	---
1418460_at	103755_at	0	Sh3d19	SH3 domain protein D19	Shimomura Y, Aoki N et al. J Derm Sci 2003
1418461_at		0	Sh3d19	SH3 domain protein D19	---
1445781_at		0.01	Sh3d19	SH3 domain protein D19	---
1449084_s_at		0	Sh3d19	SH3 domain protein D19	---
1449579_at	103813_at	1	Sh3yl1	Sh3 domain YSC-like 1	Aoki N, Ito K et al. JID 2000
1420653_at		0.51	Tgfb1	transforming growth factor, beta 1	Welker P, Foitzik K et al. Arch Dermatol Res 1997
1417455_at	102751_at	1	Tgfb3	transforming growth factor, beta 3	Welker P, Foitzik K et al. Arch Dermatol Res 1997
1425444_a_at		0.3	Tgfbr2	transforming growth factor, beta receptor II	Paus R, Foitzik K et al. JID 1997
1426397_at		1	Tgfbr2	transforming growth factor, beta receptor II	---
1443115_at		0.26	Tgfbr2	Transforming growth factor, beta receptor II	---
1421811_at	160469_at	1	Thbs1	thrombospondin 1	Schlake T, Beibel M et al. Gene Exp Patterns 2004
1450377_at		1	Thbs1	thrombospondin 1	---
1460302_at		1	Thbs1	thrombospondin 1	---
1448821_at	102666_at	1	Tyr	tyrosinase	Ermak G & Slominski A. JID 1997
1417717_a_at		1	Tyr	tyrosinase	---
1450772_at	103490_at	1	Wnt11	wingless-related MMTV integration site 11	Lin KK, Chudova D et al., PNAS 2004
