# Supplementary material for: Circadian Clock Genes Contribute to the Regulation of Hair Follicle Cycling
Source: PLoS Genet. 2009 Jul 24;5(7):e1000573. doi: 10.1371/journal.pgen.1000573 (PMC2705795; doi:10.1371/journal.pgen.1000573)
Supplement: Table S3 — Hair cycle staging of Bmal1 knockout mice (−/−) and their control littermates (+/+ and +/−). For each postnatal day (P), mice are grouped by genotype and each mouse is classified into specific phases/stages of the hair growth cycle based on the majority of hair follicles using established morphological guidelines. In general, we noted a slightly more advanced hair cycle progression in male mice; the table includes both genders which accounts for most of the variation within each genotype, but does not explain the observed differences between genotypes. Hence, for hair cycling progression comparison across genotypes, we matched littermates by gender. (0.06 MB PDF) [file pgen.1000573.s008.pdf]

Table S3

	P14			P22			P24			P28			P30-31			P34-35			P38			P44			P50		
Genotype	+/+	+/−	−/−	+/+	+/−	−/−	+/+	+/−	−/−	+/+	+/−	−/−	+/+	+/−	−/−	+/+	+/−	−/−	+/+	+/−	−/−	+/+	+/−	−/−	+/+	+/−	−/−
Total # of mice	3	6	2	1	4	5	3	6	2	5	10	4	3	5	3	1	7	3	0	2	3	2	5	2	0	3	3
Telogen				1	4	5																				3	2
Anagen I							1		2			4															
Anagen II								1							1												
Anagen IIIa							1	1			1				1												
Anagen IIIb							1	4		2	4			2	1												
Anagen IIIc											2																
Anagen IV										3	3		1	2				1									
Anagen V													2	1				2									
Anagen VI																					3						
Catagen I																	1										
Catagen II	3	5	2													1	6			1		1		1			
Catagen III		1																		1				1			1
Catagen IV																											
Catagen V																											
Catagen VI																							1				
Catagen VII																							1				
Catagen VIII																						1	3				
