# Supplementary material for: Circadian Clock Genes Contribute to the Regulation of Hair Follicle Cycling
Source: PLoS Genet. 2009 Jul 24;5(7):e1000573. doi: 10.1371/journal.pgen.1000573 (PMC2705795; doi:10.1371/journal.pgen.1000573)
Supplement: Table S4 — Hair cycle staging of Clock mutant mice (Cl/Cl) and their control littermates (+/+ and Cl+). Methodology same as Table S3. (0.06 MB PDF) [file pgen.1000573.s009.pdf]

Table S4

	P17			P21-23			P25			P27-28			P29			P34		
Genotype	+/+	*Cl*/+	*Cl*/*Cl*	+/+	*Cl*/+	*Cl*/*Cl*	+/+	*Cl*/+	*Cl*/*Cl*	+/+	*Cl*/+	*Cl*/*Cl*	+/+	*Cl*/+	*Cl*/*Cl*	+/+	*Cl*/+	*Cl*/*Cl*
Total # of mice	3	4	1	7	9	5	5	7	5	7	7	6	1	3	2	0	4	4
Telogen				7	9	5			1									
Anagen I								1	1		1	2						
Anagen II								1	2		2	2						
Anagen IIIa							1	1	1	2	1							
Anagen IIIb							1	2		1	3	1		2	1			
Anagen IIIc							3	1		3		1			1			
Anagen IV										1			1	1			1	2
Anagen V																	1	
Anagen VI																	2	2
Catagen I																		
Catagen II																		
Catagen III																		
Catagen IV																		
Catagen V																		
Catagen VI	2	1	1															
Catagen VII	1	3																
Catagen VIII																		
