# Supplementary material for: Circadian Clock Genes Contribute to the Regulation of Hair Follicle Cycling
Source: PLoS Genet. 2009 Jul 24;5(7):e1000573. doi: 10.1371/journal.pgen.1000573 (PMC2705795; doi:10.1371/journal.pgen.1000573)
Supplement: Text S1 — Probabilistic model for detection of periodic profiles. (4.01 MB PDF) [file pgen.1000573.s010.pdf]

Circadian clock genes contribute to the regulation of hair follicle cycling

Text S1: Probabilistic model for detection of periodic profiles

1 Introduction

Generic methods for detection of periodicity from temporal gene expression data such as functional sine-wave matching [Straume, 2004], hypothesis testing in frequency domain [Wichert et al., 2004, Glynn et al., 2006], and Bayesian detection [Zhou et al., 2006] are not well suited for the data set analyzed in this paper. There are unique challenges posed by the underlying biological process and the design of the experiment.

- **Masking of the periodic signal.** Some of the hair cycling genes may play a role in the initial hair follicle morphogenesis or in the injury response to hair plucking. This may result in altered expression patterns and apparent loss of periodicity at specific time points for some true hair cycle related genes.
- **Non-sinusoidal expression patterns.** Temporal patterns of hair cycling genes have been previously characterized in our earlier work [Lin et al., 2004] and many appear to be non-sinusoidal, making functional sine-wave matching ineffective.
- **Different experimental platforms.** We incorporate expression data from different generations of the Affymetrix platform: MG-U74Av2 arrays (12488 probe sets) are used to profile the first synchronous hair cycle and asynchronous time points; MG-430 2.0 arrays (45,037 probe sets) are used to profile the second synchronous and depilation-induced hair cycles.
  - **Probe set matching.** Gene identity alone is often not sufficient for matching observations from different platforms [Nimgaonkar et al., 2003], and probe composition must be taken into account when creating profiles across all three cycles.
  - **Missing data.** Only a small fraction of all available probe sets (roughly 20% of all present ones) are profiled on both platforms. For the remaining 80% of probe sets the data is available only in the second cycle and the early phases of the depilation-induced cycle. In this context, tracking similarity across the cycles becomes impossible unless the data are shared across the probe sets.
  - **Systematic differences.** Direct comparison of expression levels from different generations of arrays is inaccurate due to the systematic offsets and scaling differences [Hwang et al., 2004].
- **Unequally spaced time grid.** Different cycles are profiled on different time grids that capture critical stages of the hair cycle but are not identical across the cycles.

To address these challenges, we develop a non-parametric probabilistic model that allows us to simultaneously identify periodically expressed genes and cluster them according to their temporal profiles. The inference procedure combines evidence from all of the available data into a posterior probability of periodicity that can be used for ranking and assessment of the remaining uncertainty.

2 Generative model

It is convenient to describe the model using generative framework: how the observations could be stochastically generated according to the structure and parameterization of the model. At the highest level, we assume that the data are generated by a two component mixture model with periodic and background components. Shared parameters of these two components $\{\Phi^p, \Phi^b\}$ control characteristics common to all probe sets, such as basic patterns of periodic expression and replicate variability. A binary component indicator l_n

selects the periodic component for probe set n with probability π ($P(l_n = 1) = \pi$). This probability π is a priori unknown, and we treat it as a random variable with a conjugate Beta prior distribution:

$$\pi \sim B(\pi\,;\, a_\pi, b_\pi) \tag{1}$$

In the next two sections, we explain how the observations Y are generated by each of these two components.

2.1 Periodic component

The periodic component encodes several key assumptions: (1) a relatively small number of basic shapes of periodic expression are shared by all periodic genes; (2) one of these basic shapes gives rise to the "ideal" cycle expression for a particular probe set; (3) unless the probe set is involved in the morphogenesis or injury response, all three individual cycles are noisy copies of the corresponding time points of the ideal cycle, which allows for systematic offsets between different experimental platforms; (4) observed replicates are independent observations of the profiles within the individual cycles.

2.1.1 Notation

To help keep track of all variables, we introduce a figure and two tables. Figure A shows the graphical model associated with the periodic component using plate notation [Buntine, 1994]. Plates correspond to individual probe sets. The probe sets are conditionally independent given shared (out-of-plate) parameters. Compared to Figure 1B in the main text, the vector variables such as individual cycle profiles, replicate variances and observed values have been collapsed into single nodes in Figure A. Out-of-plate variables previously denoted by a single node are shown explicitly. Tables 1 and 2 list all variables and parameters associated with the periodic component. The first columns contain the notation for the variable, the second column shows the dimensionality of the corresponding variable, and the last column provides a brief description.

2.1.2 Shared expression patterns

The ideal cycle defines expression across $D_0 = 9$ time points which correspond to the 9 time points of the densely profiled second cycle. We use a mixture of D_0-dimensional Gaussian distributions with diagonal covariance structure, denoted by G, to represent common expression patterns in the ideal cycle. Both the number of common expression patterns and their shapes are unknown a priori, motivating us to use the Dirichlet process prior for G [Antoniak, 1974]. The Dirichlet process prior is parameterized by a centering distribution that specifies a prior for generating components of G and a scalar concentration parameter α that controls the number of components:

$$G \sim DP(\alpha, G_0)$$

We assign a conjugate Gamma prior to the concentration parameter [West, 1992] and use a conjugate Normal-inverse Gamma centering distribution [Gelman et al., 1995]:

$$\alpha \sim \Gamma(\alpha\,;\, a_\alpha, b_\alpha)$$
$$G_0 = \mathrm{NIG}\left(\nu_{G_0}, K_{G_0}, a_{G_0}, b_{G_0}\right)$$

Let (ν_k, S_k) denote the mean and variance for the kth distinct component in G and let z_n be the allocation variable denoting the component of G used to generate observation n. The ideal profile μ_n^0 is a sample from the respective Gaussian distribution:

$$\mu_n^0 | z_n \sim \mathrm{N}(\mu_n^0\,;\, \nu_{z_n}, S_{z_n})$$

The similarity of profiles within the individual cycles $\mu_n = \{\mu_n^1, \mu_n^2, \mu_n^3\}$ arises from treating them as noisy copies of the ideal cycle profile μ_n^0.

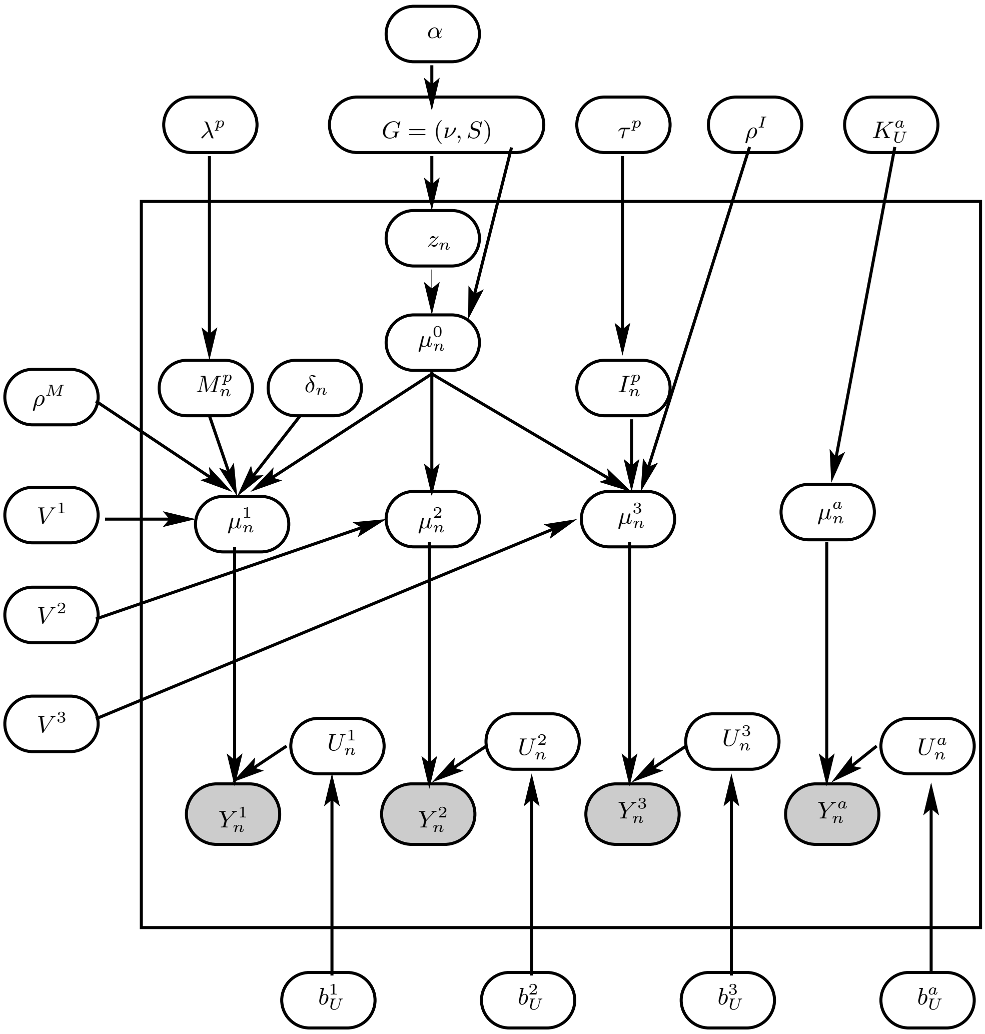

Figure A: Graphical model structure for the periodic component: $P(Y_n, \Theta^p, \Phi^p | l_n = 1)$. Variables μ_n^c and U_n^c are $|T^c|$-dimensional vectors corresponding to cycle c.

2.1.3 Time point matching

We can establish a mapping of time points within the individual cycles to the ideal cycle time line by considering the histological evaluation of the tissues. Evidence from histology, based on follicular depth and other features, reveals the actual progression through the hair cycle and helps identify the correct mapping. Figure B illustrates the resulting mapping between the cycles. Using T^c to denote the indices of the time points within the ideal cycle that correspond to the consecutive time points in cycle c, we define

$$T^1 = \{1, 3, 5, 7, 9\}; \qquad T^2 = \{1, 2, 3, 4, 5, 6, 7, 8, 9\}; \qquad T^3 = \{1, 2, 3, 4, 6\}$$

2.1.4 Cross-platform profile comparison

To compare expression levels at the corresponding time points of different cycles, we must first establish correspondence between probe sets on the different generations of the Affymetrix platform. Matching by gene identity alone is not sufficient [Nimgaonkar et al., 2003, Hwang et al., 2004, Bhattacharya and Mariani, 2005] since the individual probes within the probe sets may change from one generation to the next. We

Variable	Dim	Explanation
G	∞	Gaussian mixture model for the ideal profiles
$G_0(\nu_{G_0}, K_{G_0}, a_{G_0}, b_{G_0})$	D_0	Centering distribution of the Dirichlet process prior for G
α	1	Concentration parameter of the Dirichlet process prior for G
(ν_k, S_k)	D_0	Mean and diagonal covariance of the kth distinct component of G
$(K_\delta, a_\delta, b_\delta)$	1	Parameters of the prior distribution for offsets δ_n
λ^p	1	Probability of morphogenesis involvement
τ^p	1	Probability of injury involvement
V^c	D_c	Variance between scaled ideal cycle and scaled individual cycle c
ρ^M	$\lvert T_M \rvert$	Variance of expression levels under morphogenesis
ρ^I	$\lvert T_I \rvert$	Variance of expression levels under injury
(a_ρ^M, b_ρ^M)	$\lvert T_M \rvert$	Degrees of freedom and inverse scale for ρ^M
(a_ρ^I, b_ρ^I)	$\lvert T_I \rvert$	Degrees of freedom and inverse scale for ρ^I
(a_V^c, b_V^c)	D_c	Degrees of freedom and inverse scale for inter-cycle variability V^c
(a_U^c, b_U^c)	D_c	Degrees of freedom and inverse scale for replicate variance in cycle c

Table 1: Shared (out-of-plate) variables and fixed parameters relevant to the periodic component of the model.

use the strictest ("best match") mapping between probe sets established by the manufacturer (Affymetrix, see `http://www.affymetrix.com/support/technical/manual/comparison_spreadsheets_manual.pdf`), discarding 1120 of the probe sets that do not have a matching probe set on the newer chip. In most cases, these probe sets are superseded by improved probes on the newer chip that provide more reliable observations.

Systematic differences in observed expression intensities may exist even for carefully matched probe sets: Hwang et al. [2004] points out probe set specific differences in the absolute expression levels and the scale of measurements (confirmed in the data set studied here). In the log-transformed space, we model this by including random offset variables δ_n and allowing the profile in the first cycle to deviate from the ideal cycle μ_n^0 by δ_n (identical for all time points, but specific to probe set n).

2.1.5 Modeling the effects of morphogenesis and injury

Some of the hair cycling genes may play a role in the initial follicle morphogenesis during the first cycle or in the injury response during the depilation-induced cycle. These processes are limited to the first two time points of the corresponding cycles ($T_M = \{1, 2\}$ and $T_I = \{1, 2\}$). Consequently, affected genes may deviate from their ideal profiles at these time points. We use latent binary variables M_n^p and I_n^p to indicate involvement of probe set n in the morphogenesis and injury response respectively. For $M_n^p = 0$ and $I_n^p = 0$, the expression within individual cycles follows the ideal cycle profile; otherwise the individual cycles are independent of the ideal cycle in the affected time points.

2.1.6 Deviation between the cycles

Ignoring the effects of morphogenesis, injury response, and additive offsets, the individual cycles follow the ideal cycle profile at the corresponding time points. As demonstrated in Figures C and D, deviation of the individual cycles from the ideal cycle is proportional to the magnitude of changes in the ideal cycle.

Variable	Dim	Explanation
z_n	1	Assignment of probe set n to the component of G
μ_n^0	D_0	Ideal profile
M_n^p	1	Morphogenesis indicator
I_n^p	1	Injury indicator
δ_n	1	Additive offset between the first cycle and the ideal profile
μ_n^c	D_c	Profile within cycle c
U_n^c	D_c	Replicate variance within cycle c

Table 2: Gene-specific (in-plate) variables relevant to the periodic component of the model.

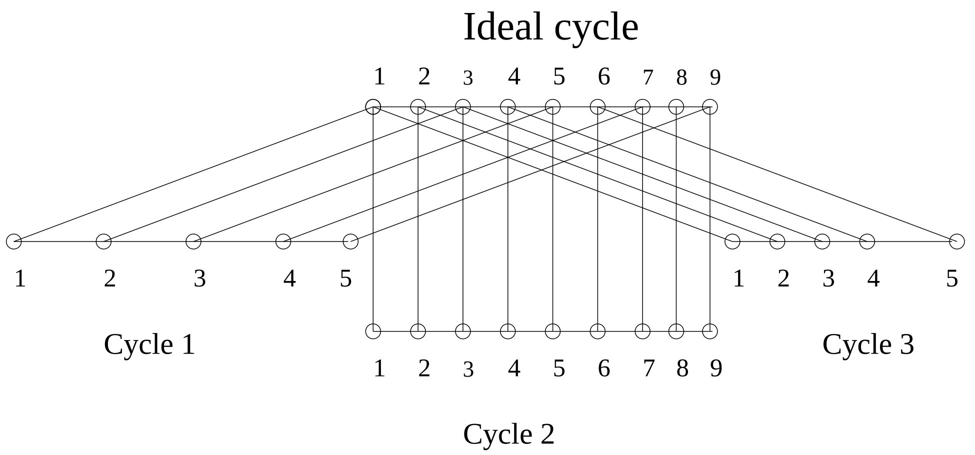

Figure B: Mapping of time points between cycles.

For these plots, we use probe sets identified previously as hair cycling based on the comparison of replicate variability in the first cycle and the asynchronous time points (p-values from Lin et al. [2004] below $1e-04$). The x-axis is the mean absolute deviation of the empirical mean profile in the second cycle. The y-axis is the absolute difference in the expression levels between the two matched time points. Figure C shows the deviation between cycles 1 and 2, and Figure D shows the deviation between cycles 3 and 2. Each subplot corresponds to a single time point within the first or the third cycle. Bold line is a *loess* fit to the data.

A strong dependence is evident in these plots: the larger the overall magnitude of changes in the second cycle, the larger differences there are between the corresponding time points of the cycles. Moreover, the exact relationship (slope) appears time-dependent. This phenomenon likely arises from the imperfect matching of time points; variation in the subjects' progression through the hair cycle results in sampling at slightly different subjective times. In profiles with high rates of change, which are most common in profiles with high overall magnitude of change, we see greater deviation among the observations. We account for these effects by including both a time-dependent variance V^c and the amplitude $A(\mu^0)$ of the mean profile in our inter-cycle deviation model, described next.

2.1.7 Similarity across different cycles: putting it all together

Now we present the final model for generating true profiles μ_n given the ideal cycle profile μ_n^0. We assign a shared Normal-inverse Gamma prior distribution to offsets δ_n:

$$P(\delta_n) \sim \mathrm{NIG}(\delta_n\,;\,0, K_\delta, a_\delta, b_\delta)$$

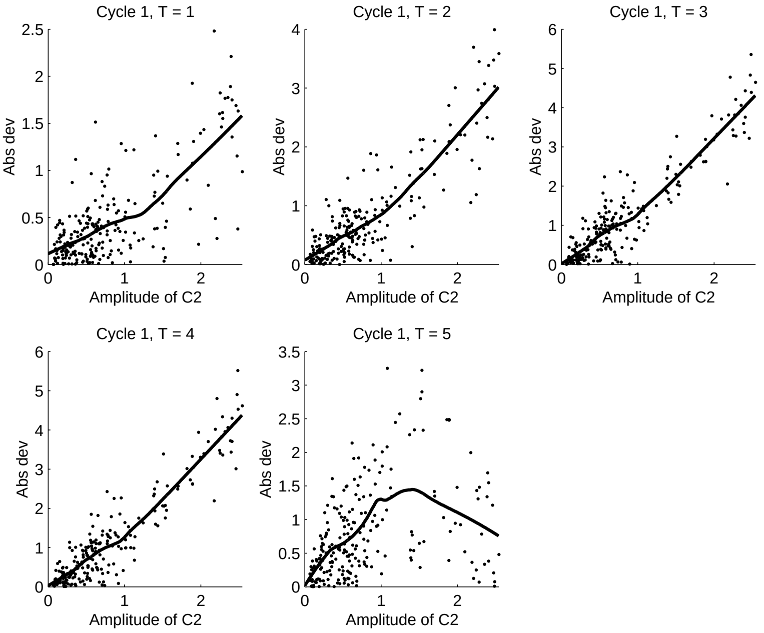

Figure C: Deviation between cycles 1 and 2 as a function of the mean absolute deviation within the second cycle for the previously identified hair-cycling genes.

Although there might be some correlation between morphogenesis and injury response, we do not expect that to be a significant factor and model them with independent Bernoulli distributions and conjugate beta priors:

$$P(M_n^p = 1) = \lambda^p, \quad P(\lambda^p) = B(\lambda^p\,;\, a_{\lambda^p}, b_{\lambda^p})$$
$$P(I_n^p = 1) = \tau^p, \quad P(\tau^p) = B(\tau^p\,;\, a_{\tau^p}, b_{\tau^p})$$

If the probe set is not involved in the morphogenesis or injury, its profile in all individual cycles follows the ideal cycle (with some offset δ_n for the first cycle):

$$P(\mu_n^1|\mu_n^0, \delta_n, M_n^p = 0) = \mathrm{N}(\mu_n^1\,;\, \mu_{n,T^1}^0 + \delta_n, V^1 \cdot A(\mu_n^0))$$
$$P(\mu_n^2|\mu_n^0) = \mathrm{N}(\mu_n^2\,;\, \mu_{n,T^2}^0, V^2 \cdot A(\mu_n^0))$$
$$P(\mu_n^3|\mu_n^0, \delta_n, I_n^p = 0) = \mathrm{N}(\mu_n^1\,;\, \mu_{n,T^3}^0, V^3 \cdot A(\mu_n^0))$$

The variance of the inter-cycle dependence $V^c \cdot A(\mu_n^0)$ scales with the variance of the ideal profile $A(\mu_n^0)$:

$$A(\mu_n^0) = \frac{1}{D_0} \sum_{j=1}^{D_0} (\mu_{nj}^0 - \bar{\mu}^0{}_n)^2$$

The variance for unit amplitude ideal cycle is given by the D^c-dimensional parameter V^c, shared across the genes. We assign a conjugate inverse Gamma prior distribution for each dimension of V^c:

$$V^c \sim \Gamma^{-1}(V^c\,;\, a_V^c, b_V^c) \qquad (2)$$

If the gene is involved in morphogenesis, it is generated independently of the ideal cycle by a Gaussian distribution with a fixed (zero) mean and random variance ρ^M in the affected time points ($T_M = \{1, 2\}$):

$$P(\mu_{n,j}^1|\mu_n^0, \delta_n, M_n^p = 1) \sim \mathrm{N}(\mu_{n,j}^1\,;\, 0, \rho_j^M,), \qquad j \in T_M$$

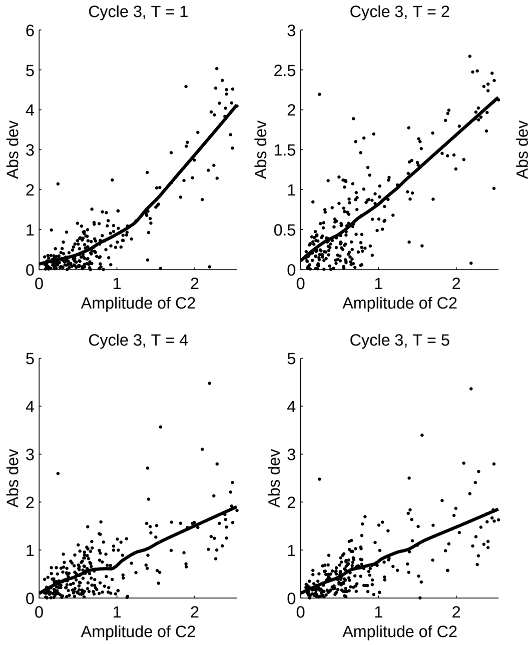

Figure D: Deviation between cycles 2 and 3 as a function of the mean absolute deviation within the second cycle for the previously identified hair cycling genes.

We fix the mean to zero as the genes can be both up-regulated and down-regulated compared to the rest of the cycle and there is no preference for positive or negative values. The variance follows an inverse Gamma prior distribution:

$$P(\rho_j^M) \sim \Gamma^{-1}(\rho_j^M\,;\, a_{\rho,j}^M, b_{\rho,j}^M), \qquad j \in T_M$$

Expression in the remaining time points follows the ideal cycle in all unaffected time points:

$$P(\mu_{n,j}^1|\mu_n^0, \delta_n, M_n^p = 1) \sim \mathrm{N}(\mu_{n,j}^1\,;\, \mu_{n,T_j^1}^0 + \delta_n, V_j^1 \cdot A(\mu_n)), \qquad j \notin T_M$$

Similarly, expression in the depilation-induced cycle is sampled independently from the ideal cycle in the affected time points and follows the ideal cycle in the remaining time points:

$$P(\rho_j^I) \sim \Gamma^{-1}(\rho_j^I\,;\,, a_{\rho,j}^I, b_{\rho,j}^I), \qquad j \in T_I$$
$$P(\mu_{n,j}^3|\mu_n^0, I_n^p = 1) \sim \mathrm{N}(\mu_{n,j}^3\,;\, 0, \rho_j^I), \qquad j \in T_I$$
$$P(\mu_{n,j}^3|\mu_n^0, I_n^p = 1) \sim \mathrm{N}(\mu_{n,j}^3\,;\, \mu_{n,T_j^3}^0, V_j^3 \cdot A(\mu_n)), \qquad j \notin T_I$$

2.1.8 Replicate Variability

Replicate variability of periodic genes U_n^c is not known a priori, and we learn it from data. We treat the degrees of freedom parameter a_U^c as a fixed quantity and assign conjugate distributions to the scale of the mean K_U^c and the inverse scale parameter b_U^c:

$$P(K_U^c) \sim \Gamma\left(K_U^c\,;\, a_{K_U}^c, b_{K_U}^c\right)$$
$$P(b_U^c) \sim \Gamma^{-1}\left(b_U^c\,;\, a_{b_U}^c, b_{b_U}^c\right)$$

2.1.9 Asynchronous time points

We treat asynchronous time points independently from the other cycles and use a Normal-inverse Gamma prior for the latent 3-dimensional profiles μ_n^a and replicate variances U_n^a:

$$P(\mu_n^a, U_n^a) \sim \mathrm{NIG}(0, K_U^a, a_U^a, b_U^a) \tag{3}$$

2.1.10 Observed data

Given latent profiles within the individual cycles μ_n^c and replicate variability U_n^c, the actual observations Y_n are assumed independent and normally distributed:

$$P(Y_{njr}^c) \sim \mathrm{N}\left(Y_{njr}^c\,;\,\mu_{nj}^c, U_{nj}^c\right), \quad c \in \{1,2,3,a\}, 1 \le j \le D_c;\,;\; 1 \le r \le R_{nj}^c$$

2.2 Background component

Unless non-periodic genes are involved in the morphogenesis or injury response, they remain constant within the cycles. Similarly to the periodic component, we model morphogenesis indicators M_n^b and injury response indicators I_n^b as independent binary variables with conjugate beta priors:

$$P(M_n^b = 1|\lambda^b) = \lambda^b, \quad P(\lambda^b) = B(\lambda^b\,;\,a_{\lambda^b}, b_{\lambda^b})$$
$$P(I_n^b = 1|\tau^b) = \tau^b, \quad P(\tau^b) = B(\tau^b\,;\,a_{\tau^b}, b_{\tau^b})$$

We will introduce a short-hand notation and say that (x, σ) are distributed as sNIG_j^D if they follow a singular D-dimensional Normal-inverse Gamma prior where dimensions $1\ldots j+1$ are all independent, while dimensions $j+1\ldots D$ are constrained to be equal:

$$P(\sigma) = \prod_{q=1}^{j+1} \Gamma^{-1}(\sigma_q\,;\,a_q, b_q) \prod_{q=j+2}^{D} \delta(\sigma_q - \sigma_{j+1})$$
$$P(x|\sigma) = \prod_{q=1}^{j+1} \mathrm{N}(x_q\,;\,\nu_q, \sigma_q/K_q) \prod_{q=j+2}^{D} \delta(x_q - x_{j+1})$$

where $\delta(x)$ is the Dirac delta function.

Let η_n^c and W_n^c denote the vectors for true expression levels and replicate variances for probe set n within cycle c. Expression in the first and the third cycles depends on the values of M_n^b and I_n^b, respectively, and we use a second superscript on the distribution parameters to denote the value of the corresponding indicator.

In the first cycle, the true expression remains constant when the gene is not involved in the morphogenesis:

$$P(\eta_n^1, W_n^1|M_n^b = 0) = \mathrm{sNIG}_0^{D_1}\left(\eta_n^1, W_n^1\,;\,\eta_0^{1,0}, K_W^{1,0}, a_W^{1,0}, b_W^{1,0}\right) \tag{4}$$

If $M_n^b = 1$, the expression can vary independently at the time points in T_M:

$$P(\eta_n^1, W_n^1|M_n^b = 1) \sim \mathrm{sNIG}_{\max\{T_M\}}^{D_1}\left(\eta_n^1, W_n^1\,;\,\eta_0^{1,1}, K_W^{1,1}, a_W^{1,1}, b_W^{1,1}\right) \tag{5}$$

Expression in the second hair cycle is constant irrespective of M_n^b and I_n^b:

$$P(\eta_n^2, W_n^2) \sim \mathrm{sNIG}_0^{D_2}\left(\eta_n^2, W_n^2\,;\,\eta_0^2, K_W^2, a_W^2, b_W^2\right) \tag{6}$$

In the third cycle, the true expression is constant when the gene is not involved in the injury response:

$$P(\eta_n^3, W_n^3|I_n^b = 0) \sim \mathrm{sNIG}_0^{D_3}(\eta_n^3, W_n^3\,;\,\eta_0^{3,0}, K_W^{3,0}, a_W^{3,0}, b_W^{3,0}) \tag{7}$$

If $I_n^b = 1$, the expression can vary independently at the time points in T_I:

$$P(\eta_n^3, W_n^3 | I_n^b = 1) \sim \mathrm{sNIG}^{D_3}_{\max\{T_I\}} \left(\eta_n^3, W_n^3\, ;\, \eta_0^{3,1}, K_W^{3,1}, a_W^{3,1}, b_W^{3,1}\right) \tag{8}$$

Finally, the true expression levels and replicate variances in the asynchronous time points are independent across time points and generated by a 3-dimensional NIG prior:

$$P(\eta_n^a, W_n^a) \sim \mathrm{NIG}\left(\eta_n^a, W_n^a | \eta_0^a, K_W^a, a_W^a, b_W^a\right) \tag{9}$$

The amount of replicate variability for background genes is controlled by the number of degrees of freedom and the inverse scale (a, b) of the inverse Gamma distributions in Equations (4) through (9). These parameters are a priori unknown and we learn them from data by assigning a conjugate inverse Gamma prior to the inverse scale b, shared across all genes:

$$P(b_W^{c,x}) \sim \Gamma^{-1}\left(b_W^{c,x}\, ;\, a_{b_W^{c,x}}, b_{b_W^{c,x}}\right)$$

where x stands for the value of the morphogenesis and injury indicators. The degrees of freedom parameter a is treated as a fixed quantity in this version of the model.

The actual observed expression values for all time points are generated independently by a Gaussian distribution with mean η_n and replicate variance W_n:

$$P(Y_{njr}^c | \eta_n, W_n) = \mathrm{N}(Y_{njr}^c | \eta_{nj}^c, W_{nj}^c), \qquad 1 \le r \le R_{nj}^c$$

This completes the specification of the dependency structure and conditional probability distributions associated with the model variables. To complete the model specification, we next provide the parameter values for the prior models, and turn to developing an inference procedure for estimating the posterior distribution over the relevant model variables given observed data Y.

2.3 Specifying prior distributions

We can use our knowledge of genes likely to be periodic to aid in specifying the prior distributions for the model. A previous computational analysis of hair cycling genes has been carried out using the subset of data corresponding to the first cycle and asynchronous time point measurements [Lin et al., 2004]. The method used was quite different from the proposed model and relied on comparing replicate variability within the first hair cycle and asynchronous time points. Using the set of tentative hair cycling genes from that analysis to construct the priors for the model is not, strictly speaking, "proper" as it uses some of the data twice, but may be viewed as an empirical Bayes approximation. Since we only use these results to estimate the parameters of the hyperprior distributions for replicate variance parameters, this should not have a sizable effect.

Based on previous analysis we expect approximately one third of all present probe sets to be periodic. We set the parameters of the Beta distribution for the probability of periodic component π as $(a_\pi, b_\pi) = (1000, 2000)$, so that the strength of the prior is roughly equivalent to 10% of the entire data set.

Similarly, we expect between $5\% - 10\%$ of the genes to be involved in morphogenesis and injury response and thus, use the following priors:

$$(a_{\lambda^p}, b_{\lambda^p}) = (25, 475), \quad (a_{\tau^p}, b_{\tau^p}) = (25, 475)$$
$$(a_{\lambda^b}, b_{\lambda^b}) = (25, 475), \quad (a_{\tau^b}, b_{\tau^b}) = (25, 475)$$

2.3.1 Periodic component

We use a weakly informative prior for the Dirichlet process mixture components with low degrees of freedom for the component variance and low scale for the mean: $\mathrm{NIG}(\nu_{G_0}, K_{G_0}, a_{G_0}, b_{G_0}) = \mathrm{NIG}(0, 0.1, 2, 0.2)$. The

scale of the mean influences the prior's strength; we set it to a low (relatively uninformative) value. We also use a weak Gamma prior for the concentration parameter of the Dirichlet process prior: $(a_\alpha, b_\alpha) = (1e-10, 1e-10)$.

The offset between the cycles is expected to vary around zero, $\delta \sim \text{NIG}(0, 1, 2, 2)$. The inter-cycle variability V is assigned a prior that encourages lower values $(a_V, b_V) = (2, 0.1)$. Parameters (a_ρ, b_ρ) of the inverse Gamma prior for the variance of expression levels in the morphogenesis- or injury–affected time points are selected so that the mean of the prior equals the variance of expression levels for the top 10% highly varying genes in these time points, resulting in $(a_\rho^M, b_\rho^M) = (75.3902, 201.5280)$ and $(a_\rho^I, b_\rho^I) = (103.853, 328.2509)$.

Finally, we find the maximum likelihood estimates of the parameters of the replicate variance distribution within each time point $(a_{U,j}^c, b_{U,j}^c)$, where the observed data are the empirical estimates of replicate variance for the set of genes previously identified as hair cycling ($p < 0.001$ in Lin et al. [2004]). The degrees of freedom parameter $a_{U,j}^c$ is kept fixed, and the inverse scale $b_{U,j}^c$ is assigned inverse Gamma prior with mean value equal to the maximum likelihood estimate $b_{U,j}^c$ and variance $0.5 * b_{U,j}^c$.

For the asynchronous time points, we use the same process to set the degrees of freedom of the replicate variance distribution a_U^a and parameters of the prior for the inverse scale $(a_{b_U}^a, b_{b_U}^a)$. The scale of the mean K_U^a is assigned a Gamma prior that encourages lower values of the scale, $(a_{K_U}^a, b_{K_U}^a) = (0.1, 1)$.

2.3.2 Background component

In the background component, we set the location of the mean $\eta_0^{c,x}$ to 0 for all cycles c and all values of morphogenesis and injury indicators x. Replicate variability does not depend on the morphogenesis and injury indicators, so we use the same prior distributions in all cases of M^b and I^b. Similarly to the method used for the periodic component, we begin by finding maximum likelihood estimates of parameters $(a_W^{c,x}, b_W^{c,x})$, where the observed data are the empirical estimates of replicate variability for each of the genes previously not identified as hair cycling ($p > 0.1$). The number of degrees of freedom is then kept fixed, and the inverse scale is assigned a prior distribution with the mean equal to the estimated $b_W^{c,x}$ and variance $0.5 b_W^{c,x}$.

Parameters of the prior for the location of the mean $(a_{K_{W,j}}^{c,x}, b_{K_W}^{c,x})$ are shown in table 3 as they are specific to the cycle and the values of binary indicators. We set these parameters by matching the mean and variance values given in the fourth and fifth columns of the table. In general, larger values of the scale parameter K correspond to smaller variance in the mean profile η.

Cycle	Indicator	Time points	Mean	Variance	Parameters
c = 1	$M^b = 0$	$j \in [1 \dots D_1]$	100	100	(100,1)
c = 1	$M^b = 1$	$j \in T_M$	0.05	1	(0.0025,20)
c = 1	$M^b = 1$	$j \notin T_M$	1	10	(0.01,10)
c = 2		$j \in [1 \dots D_2]$	100	100	(100,1)
c = 3	$I^b = 0$	$j \in [1 \dots D_3]$	0.5	0.5	(0.5,1)
c = 3	$I^b = 1$	$j \in T_I$	0.5	0.5	(0.5,1)
c = 3	$I^b = 1$	$j \notin T_I$	0.5	0.5	(0.5,1)
c = a		$j \in [1 \dots D_a]$	0.1	0.1	(0.1,1)

Table 3: Parameters of the prior for the scale of the mean

3 Inference

The exact inference over model parameters given data Y is not possible for this model, and we use approximation schemes to infer the indicators of periodicity l_n for each of the probe sets as well as cluster assignment of profiles within the periodic component. The standard Gibbs sampling approach has very low convergence rate in this model due the switching variable l_n that chooses one of the two alternative explanations for Y_n

1 **function** $\left(\Phi^{(new)}, \Theta^{(new)}\right) \leftarrow \text{HCGibbsIter}(Y, \Phi, \Theta)$
2 $\Phi^{(new)} \leftarrow \Phi;$
3 $\Theta^{(new)} \leftarrow \Theta;$
4
5 **for** all $\phi \in \Phi$
6 $\quad \phi^{(new)} \sim P(\phi | Y, \{\Phi^{(new)} \setminus \phi\});$
7 **end**
8
9 **for** n $\leftarrow$ 1 : N
10
11 $\quad {l_n}^{(new)} \sim P(l_n | Y_n, \Phi^{(new)});$
12
13 $\quad {\Theta_n^p}^{(new)} \sim P(\Theta_n^p | Y_n, \Phi^{(new)}, {l_n}^{(new)});$
14 $\quad {\Theta_n^b}^{(new)} \sim P(\Theta_n^b | Y_n, \Phi^{(new)}, {l_n}^{(new)});$
15
16 **end**

Table 4: High-level description of the blocked Gibbs sampler for the model for identification of periodic genes.

(periodic or background component). Given the value of the indicator l_n, the conditional posterior for one of these two explanations for Y_n is independent of Y_n and coincides with the prior. Sampling from the prior makes changing the indicator value l_n very unlikely at subsequent iterations.

We can address this problem using blocking [Liu et al., 1994] to sample the highly correlated component indicators and gene-specific variables in a single update. Table 4 provides high-level pseudo-code for a single iteration of the blocked sampler: we alternate (1) sequential updates for all shared variables $\Phi = \{\pi, \Phi^p, \Phi^b\}$ and (2) joint updates for all gene-specific variables and component indicators $\Theta = \{\{l, \Theta^p, \Theta^b\}_{n=1}^N\}$.

In lines 5 through 7 we sequentially update all out-of-plate (shared) parameters using standard conjugate updates:

$$\Phi = \{\Phi^p, \Phi^b\} = \{\{\alpha, (\nu_k, S_k)_{k=1}^K, \lambda^p, \tau^p, \rho^M, \rho^I, V, b_U, K_U^a\}, \{b_{b_W}, \lambda^b, \tau^b\}\}$$

In lines 9 through 16 we update gene-specific parameters for each of the N probe sets. The joint distribution of $\Theta_n = \{l_n, \Theta_n^p, \Theta_n^b\}$ can be factorized as

$$P(l_n, \Theta_n^p, \Theta_n^b | Y_n, \Phi) = P(l_n | Y_n, \Phi) P(\Theta_n^p, \Theta_n^b | Y_n, \Phi, l_n)$$

and the samples from the joint are obtained in two steps:

$$l_n \sim P(l_n | Y_n, \Phi)$$
$$\Theta_n^p, \Theta_n^b \sim P(\Theta_n^p, \Theta_n^b | Y_n, \Phi, l_n)$$

First, we sample component assignment l_n from its marginal posterior distributions (line 11, $\{\Theta_n^p, \Theta_n^b\}$ are integrated out). After that, $\{(\Theta_n^p, \Theta_n^b\}$ can be sampled from their conditional distributions (lines $13, 14$).

Marginal component probability $P(l_n | Y_n, \Phi)$ does not allow closed-form expressions as it requires evaluating marginal likelihood of observations Y_n when gene-specific parameters Θ_n^p are integrated out. We adapt approximate methods proposed by Chib [1995] and Chib and Jeliazkov [2001] that use the trace of

the MCMC simulation from the posterior distribution $P(\Theta_n^p|Y_n, \Phi^p)$ to evaluate marginal likelihood. This step requires running a separate MCMC chain for each of the probe sets given the current set of shared parameters Φ^p, resulting in a relatively high computational cost.

To estimate the posterior probability of periodicity for each of the probe sets, we simply average conditional posterior estimates of l_n obtained at each iterations:

$$P(l_n|Y) = \sum_{g=1}^{G} P(l_n|Y, \Phi^{(g)}) \tag{10}$$

where G is the total number of iterations and $\Phi^{(g)}$ are the actual samples of shared parameters.

4 Model application and performance

4.1 Data preprocessing

In this section, we explain how the observations Y are derived from the expression values generated by the experimental platform. This process is illustrated in the diagram in Figure E. We use the Affymetrix software suit MAS 5.0 (`http://www.affymetrix.com/products/software/specific/mas.affx`) to generate probe set summary expression values from the individual probes and a flag indicating the reliability of the intensity estimate (present call, absent call, marginal). There are 12488 probe sets on the MG-U74Av2 chip used to profile the first cycle and the asynchronous time points, and 45037 probe sets on the MG-430 2.0 chip. This is shown by the two blocks in the top of Figure E.

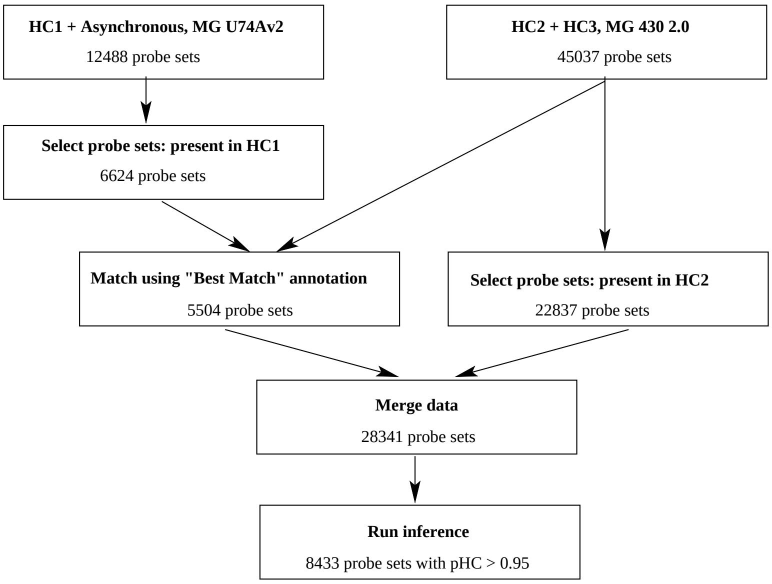

Figure E: A schematic view of the data processing flow in the data analysis.

In general, the expression estimates are affected by additive and multiplicative errors [Huber et al., 2003]. Using the two-component noise model, Rocke and Durbin [2001], Huber et al. [2002], and Durbin et al. [2002] derive an appropriate variance stabilizing transformation that can be used to achieve signal-independent errors. We estimate parameters of the transformation separately for the data from each of two different platform generations using an algorithm described in Geller et al. [2003].

After the transformation, we filter out genes that are absent during the entire cycle according to MAS 5.0 calls. From the experiments on the older platform, we retain only probe sets for which all replicates are

present in at least a single time point of the first hair cycle (6624 probe sets). Of these, we can match 5504 probe sets to the newer chip using the "Best Match" annotations (`http://www.affymetrix.com/support/technical/manual/comparison_spreadsheets_manual.pdf`). Of the remaining probe sets on the newer platform, we select 22837 probe sets for which all replicates are present in at least one time point of the second hair cycle. For these probe sets, the data corresponding to the first cycle and the asynchronous time points are treated as missing. The filtering and probe set matching steps result in 28341 probe sets that are included in the analysis.

When clustering the patterns of expression over time, we are mostly interested in finding common shapes, irrespective of the absolute values. We can not simply normalize the expression levels to zero mean within each cycle independently as (1) the cycles are profiled on different time grids, (2) the third cycle is profiled only partially, and (3) morphogenesis and injury involvement can change the average within the cycle. We normalize the expression to zero-mean independently within each experimental platform: the latent offsets δ_n between experimental platforms can compensate for the effects of such normalization.

Specifically, let Z^c_{njr} to denote the transformed intensity values for the rth replicate of gene n in the jth time point of cycle c. Then, the corresponding data Y analyzed by the model are obtained by subtracting the average of all observations within the cycle:

$$\begin{aligned}
m^1_n &= \frac{\sum_{j=1}^{D_1} \sum_{r=1}^{R^1_{nj}} Z^1_{njr}}{\sum_{j=1}^{D_1} R^1_{nj}} \\
m^2_n &= \frac{\sum_{j=1}^{D_2} \sum_{r=1}^{R^2_{nj}} Z^2_{njr}}{\sum_{j=1}^{D_2} R^2_{nj}} \\
Y^1_{njr} &= Z^1_{njr} - m^1_n \\
Y^2_{njr} &= Z^2_{njr} - m^2_n \\
Y^3_{njr} &= Z^3_{njr} - m^2_n \\
Y^a_{njr} &= Z^a_{njr} - m^1_n
\end{aligned}$$

We can now analyze the transformed and normalized data Y in order to identify periodic genes and extract their common expression patterns.

4.2 Running the inference

Since every iteration of the blocked Gibbs sampler over the entire data set is computationally expensive, we attempt to initialize in a region of high posterior density. This can be done in two steps: (1) sensibly selecting some initial parameter values and then (2) running the Gibbs sampler on a subset of the data to move into the high posterior density region. Final values of the shared parameters and periodicity indicators are then used to initialize the run on the full data.

Specifically, we initialize $\pi = 0.3$, $\lambda^p = 0.05$, $\tau^p = 0.05$, $\lambda^b = 0.05$, $\tau^b = 0.05$, $V = 0.1$. All other shared parameters, such as location of the means and scale of replicate variance distributions, are initialized to their mean values. The components of the mixture model G are initialized by learning a finite mixture model with $K = 20$ clusters on the second cycle profiles of the genes previously identified to be hair cycling. The initialization of gene-specific parameters is not important as they are are integrated out during the estimation of periodicity indicators.

This results in multiple initializations for the full run, and we can perform the full run in parallel on multiple computers as well. In practice, we have run the blocked sampler for a total of 1600 iterations over multiple (7) shorter chains, integrating out gene-specific parameters at each iteration with internal MCMC chains of length 1000 samples for each of the probe sets. The variables of greatest interest to us are the indicators of periodicity l_n: we would like the inference to converge with respect to their estimates.

In Figure F we compare the ranking of probe sets according to the values of $P(l_n|Y)$ as estimated by two such chains. The results for other Gibbs chains are highly similar. On each of the four subplots, the x and

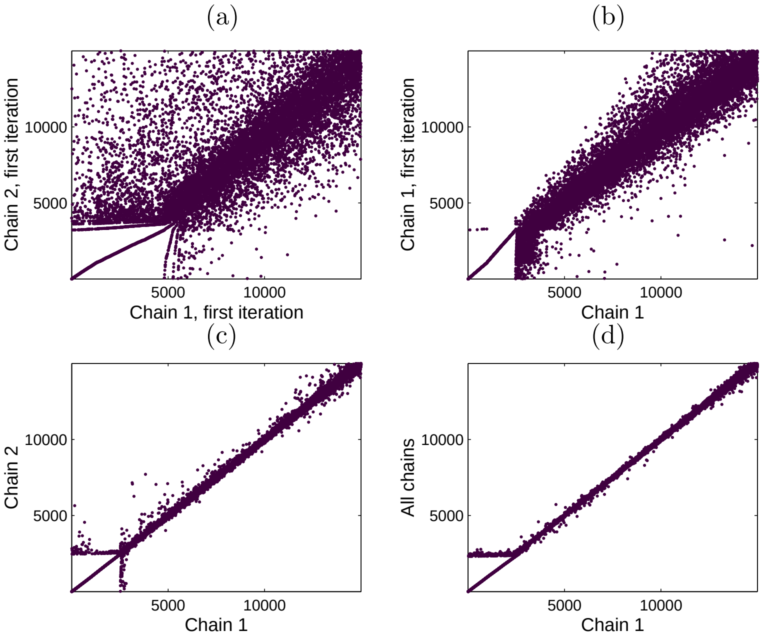

Figure F: Comparing ranking by the posterior probability of periodicity estimated by different Gibbs sampling chains. (a) Comparing two initializations; (b) Comparing an initialization with its final ranking; (c) Comparing final rankings for two runs; (d) Comparing rankings from one run with the ranking obtained by averaging all runs.

y axis are the ranking positions (up to 15000) obtained for a given probe set using the method indicated in the axis labels. If the two rankings fully agreed with each other, all of the dots would fall on the diagonal line. [1]

In panel (a), we compare the rankings implied by the first iteration of the Gibbs samplers that use two different initial values for shared parameters (obtained by running on the subsets of the data). Although there is general agreement between them, especially on the high-ranking genes, there are a large number of genes for which rankings differ significantly. Panel (b) shows the ranking implied by a single short chain versus the initial values for the same chain. However, when we use all iterations within two short Gibbs chains to compute the estimates ($G \approx 200$), the rankings are highly similar [panel (c)]. Finally, we can average out the differences between different short chains by pooling together the conditional posteriors from all of them. The plot on the bottom right shows the ranking from a single short chain versus the ranking obtained by averaging the posterior probabilities from all chains. Although only a small number of iterations is being used to estimate the indicators, the marginalization of gene-specific parameters within each iteration allows the sampler to converge to a stable ranking.

4.3 Identification of the periodically expressed probe sets

In addition to estimating the mean posterior probability of periodicity, we evaluate $[10\%, 90\%]$ confidence intervals for l_n as the $10\%^{th}$ and $90\%^{th}$ percentile of $P(l_n|Y, \Phi^{(g)})$ over all $g = 1, \ldots, G$. Figure G shows the estimates of $P(l_n|Y)$ along with the confidence intervals for the entire data set. The bold solid line represents the sorted estimates of $P(l_n|Y)$, and vertical lines show the confidence intervals for each of the probe sets. There is little variance in the estimates for the highest ranking ≈ 9000 and lowest ranking ≈ 12000 probe sets with posterior probabilities above 0.9 and below 0.1. High variability in the middle tier has contributions from both the inherent uncertainty in the value of the posterior probability and the approximate nature of

[1] An artifact can be seen near the origin of these plots due to numerical precision issues in many of the probe sets with posterior probability very close to one.

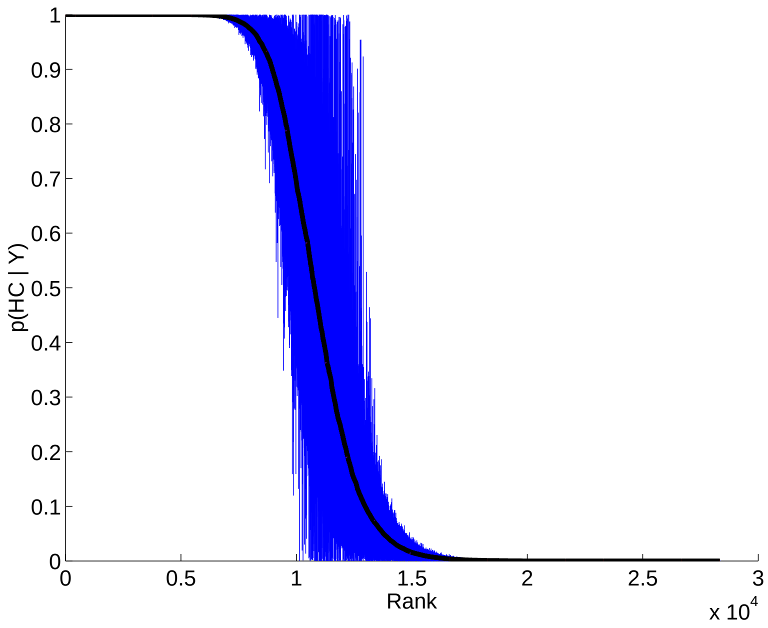

Figure G: Posterior probability of the periodic component $P(l_n|Y)$ and $[10\%, 90\%]$ confidence intervals.

the conditional posterior probability estimates. For further analysis, we will concentrate on the top ranking 8433 probe sets with $P(l_n|Y) \geq 0.95$ as these probe sets are assigned to the periodic component with high confidence.

To estimate the number of false positive discoveries in the set of the periodic probe sets, we can compute the false discovery rate (FDR): the expected fraction of true background probe sets among the probe sets that we identified as periodic. Given the posterior probabilities for all probe sets, the FDR can be computed directly by averaging the posterior probability of the background component for the selected set of top ranking probe sets Newton et al. [2004]. The FDR estimate corresponding to a threshold of 0.95 on the posterior probability is less than 5%. These estimates are necessarily optimistic as they are conditioned on the model being an accurate representation for the observed process.

To evaluate the model ranking and selection of periodic genes, we compare the performance of the model to two simpler methods that could in principle be used to identify relevant genes: identification of differential expression in the second hair cycle and identification of periodic profiles across all three cycles. The data in the second hair cycle are available for all probe sets, so we can use conventional methods, such as the two component mixture of free-varying and constant-over-time models for identification of differential expression. We use the LIMMA implementation of the model [Smyth, 2004] in the Bioconductor package (`http://www.bioconductor.org`). Since all periodic genes should exhibit non-constant expression patterns in the second hair cycle, identification of differential expression in that time period can serve as a baseline method. Naturally, this method has limitations in the context of our experiment as it is not designed to take into account the similarity across the cycles. Since the data for the great majority of probe sets are only available in the second and the beginning of the third cycle, where periodicity is possibly masked by the depilation response, the baseline solution through analysis of differential expression in the second cycle is not a bad approximation. Due to the effects of morphogenesis and injury there is no simple way to implement a more powerful search with conventional tools. The ground truth about actual hair cycling genes is not known, and we use a partial list of genes known to be regulated by the hair cycle from previous small scale experiments. We have compiled a list of 92 such genes, including 84 genes (154 probe sets) that were identified as present in at least one cycle and analyzed by the model. The number of probe sets is greater than the number of genes as some are represented by multiple probe sets. A complete set of matching probe sets

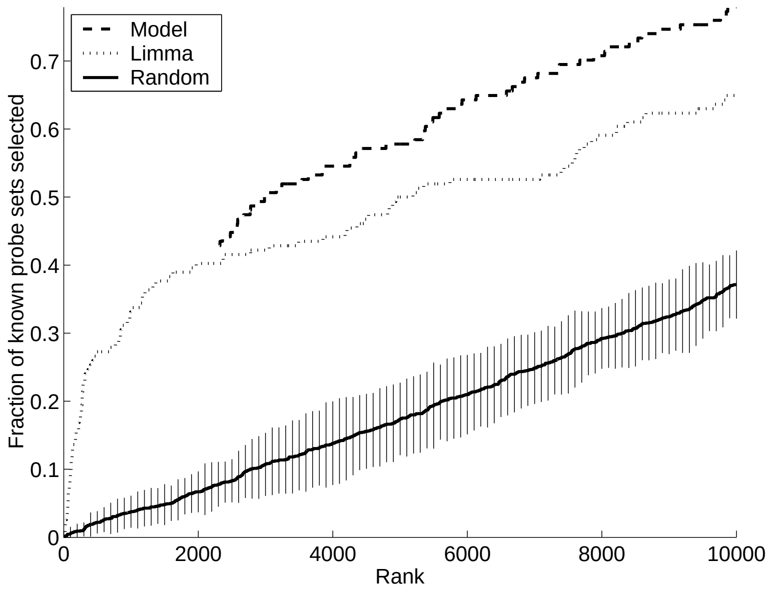

Figure H: Comparing the selection of genes known to be associated with the hair cycle by the conventional methods and the model

is given in Tables 5, 6, 7, and 8. Probe set identifiers from the newer MG-430 2.0 chip are given in the first column, followed by the identifiers on the older MG-U74Av2 platform (where available). Only 69 probe sets are profiled on both platforms. The next column contains the estimated posterior probability of periodicity. While the estimates of periodicity for the probe sets corresponding to the same gene generally agree, some probe sets have drastically different estimates. Visual examination of the corresponding profiles generally agrees with the inferred estimates of the posterior, and we attribute this discrepancy to the inefficiency of some of the probes and various technical errors rather than inherent ambiguity in the observed data for high-ranking probe sets. Columns four and five contain gene symbols and truncated gene names, and the last column provides references used to identify genes in this benchmark set.

Although these genes have been previously identified in the literature as hair cycling, there is still a varying degree of confidence in assigning them to this category. We expect a great fraction, but not all of them, to have periodic expression patterns consistent with hair cycle regulation in our data set. In Figure H, we show the fraction of the known probe sets included in the top probe sets from the ranked list, as the size of the list increases. We compare three ranking schemes: model ranking according to the posterior probability of periodicity, LIMMA ranking based on the differential expression and random ranking. In this figure, the x-axis is the number k of probe sets selected from the top of the ranked list, and the y-axis is the fraction of all benchmark probe sets among the top k ones. The line corresponding to the model does not start at $k = 1$ as all of the probe sets with posterior probability estimated as 1 are assigned the same rank. The solid line shows an average curve for the random ranking with vertical error bars corresponding to 2 standard deviations. The model consistently identifies between 10 and 20 additional probe sets at any given cut-off for the ranked list above 3000. The model selects 77 benchmark probe sets at $k = 3000$, and LIMMA reaches the same level at $k = 5000$.

Ranking according to the assessment of periodicity is another relevant baseline method. This can be meaningfully done only for the 5504 probe sets that are observed across all three partial cycles, and we illustrate the results in Figure I. Specifically, we compare our model to the ranking produced by the Lomb-Scargle periodogram for non-equidistant time grids [Glynn et al., 2006], using possible lengths of 22, 23, and 24 days. We artificially assign time stamps to the data points so that the time points are aligned as shown in Figure 1A, rather than using the biological age of the mice. This ensures that similar phases of

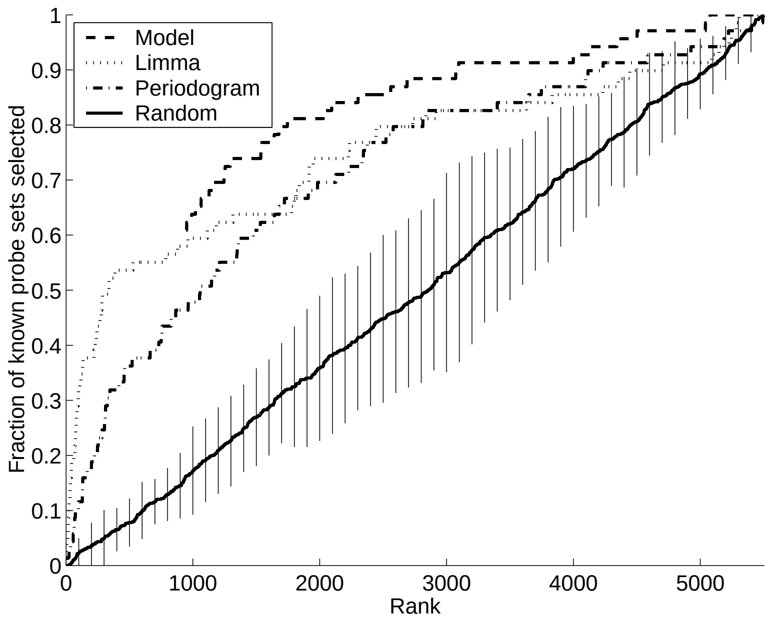

Figure I: Ranking of genes known to be associated with the hair cycle from small scale biological experiments. In this figure, we project the results to the set of genes observed across all three cycles (e.g., those common to both Affymetrix chips) in order to compare with the periodogram approach.

the cycle map to the same point on the time axis of the cycle. For comparison, we plot the results for the proposed model, LIMMA analysis, and random ranking for the 5504 probe sets. We use a "projection" of model ranking obtained from the full data set on this subset of probes rather than running the model on this subset only.

The periodogram identifies only a small fraction of probe sets as periodic (55 have p-values below 0.01, without any corrections for multiple hypothesis testing). At the same time, the model identifies 1899 genes as having posterior probability of periodicity above 0.9. Visual examination of the probes suggests that the model estimate is more accurate than what would be identified by the periodogram. The periodogram also performs worse than LIMMA in identifying the benchmark probe sets, and we speculate that it is due to (1) non-sinusoidal periodic patterns and (2) imperfect correlation across the cycles due to approximate time point matching, high replicate variability, as well as the effects of morphogenesis and injury response.

5 Conclusions

In this supplement, we have developed a Bayesian probabilistic framework for analyzing a collection of experiments profiling the hair growth cycle, based on two natural cycles and a partial artificial cycle induced by hair plucking. While some of the standard methods for the analysis of gene expression data could be applied to this data, none of them accurately reflect the reality of the experimental setup.

The proposed model allows us to simultaneously identify periodic genes and extract patterns of cyclic expression. Sharing common periodic expression profiles across the genes, implemented by a mixture model with a Dirichlet process prior, is especially valuable in this data set where the expression levels are unavailable for the entire first hair cycle for the majority of the genes. Due to the common dependence on the mixture parameters, the posterior probability of periodicity for each of the genes depends on the entire observed data rather than on a single gene's expression profile. Clustering of cycling profiles within the Dirichlet process mixture model eliminates the need for a two-step procedure that selects the periodic set followed by clustering of the selected profiles. Inference over model parameters via blocked Gibbs sampling and approximation of

the marginal component likelihood provides a principled way of combining all of the available evidence from the data.

References

C. Antoniak. Mixture of Dirichlet processes with applications to Bayesian nonparametric problems. *The Annals of Statistics*, 2(6):1153–1174, 1974.

S. Bhattacharya and T.J. Mariani. Transformation of expression intensities across generations of affymetrix microarrays using sequence matching and regression modeling. *Nucleic Acids Research*, 33(18), 2005.

W. Buntine. Operations for learning with graphical models. *Journal of the Artificial Intelligence Research*, 2:159–225, 1994.

S. Chib. Marginal likelihood from the Gibbs output. *Journal of the Americal Statistical Association*, 90: 1313–1321, 1995.

S. Chib and I. Jeliazkov. Marginal likelihood from the Metropolis-Hastings output. *Journal of the Americal Statistical Association*, 96:270–281, 2001.

B.P. Durbin, J.S. Hardin, D.M. Hawkins, and D.M. Rocke. A variance-stabilizing transformationfor gene-expression mixroarray data. *Bioinformatics*, 18(Suppl.1):S105–S110, 2002.

S.C. Geller, J.P. Gregg, P. Hagerman, and D.M. Rocke. Transformation and normalization of oligonucleotide microarray data. *Bioinformatics*, 19(14):1817–1823, 2003.

A. Gelman, J. B. Carlin, H. S. Stern, and D. B. Rubin. *Bayesian Data Analysis.* Chapman & Hall, New York, NY, 1995.

E. F. Glynn, J. Chen, and A.R. Mushegian. Detecting periodic patterns in unevenly spaced gene expression time series using lombscargle periodograms. *Bioinformatics*, 22(3):310–316, 2006.

W. Huber, A. von Heydebreck, H. Sltmann, A. Poustka, and M. Vingron. Variance stabilization applied to microarray data calibratrion and to quantification of differential expression. *Bioinformatics*, 18, 2002.

W. Huber, A. von Heydebreck, and M. Vingron. Analysis of microarray gene expression data. 2003.

K.B. Hwang, S.W. Kong, S.A. Greenberg, and P.J.Park. Combining gene expression data from different generations of oligonucleotide arrays. *BMC Bioinformatics*, 5(159), 2004.

K.K. Lin, D. Chudova, G.W. Hatfield, P. Smyth, and B. Andersen. Identification of hair cycle-associated genes from time-course gene expression profile data by using replicate variance. *PNAS*, 101(45):15955–15960, 2004.

J. Liu, W. Wong, and A. Kong. Covariance structure of the Gibbs sampler with applications to the comparison of estimators and augmentation schemes. *Biometrika*, 81(1):27–40, 1994.

M.A. Newton, A. Noueiry, D. Sarkar, and P. Ahlquist. Detecting differential gene expression with a semi-parametric hierarchical mixture model. *Biometrics*, 5:155–176, 2004. EBarrays and derivation of the FDR based on the posterior probabilities.

A. Nimgaonkar, D. Sanoudou, A.J.Butte, J.N. Haslett, L.M. Kunkel, A.H. Beggs, and I.S. Kohane. Reproducibility of gene expression across generations of affymetrix microarrays. *BMC Bioinformatics*, 4(27), 2003.

D.M. Rocke and B. Durbin. A model for measurement error for gene expression analysis. *Journal of computational biology*, 8:557–569, 2001.

G. K Smyth. Linear models and empirical bayes methods for assessing differential expression in microarray experiments. *Statistical Applications in Genetics and Molecular Biology*, 3(1), 2004.

M. Straume. *dna* microarray time series analysis: Automated statistical assessment of circadian rhythms in gene expression patterning. *METHODS IN ENZYMOLOGY*, 383:149–166, 2004.

M. West. Hyperparameter estimation in Dirichlet process mixture models. *Technical Report 92–A03, Institue of Statistic and Decision Sciences, Duke University*, 1992.

S. Wichert, K. Fokianos, and K. Strimmer. Identifying periodically expressed transcripts in microarray time series data. *Bioinformatics*, 20(1):5–20, 2004.

Chuan Zhou, Jon C Wakefield, and Linda L Breeden. Bayesian analysis of cell cycle gene expression data. In Kim-Anh Do, Peter Müller, and Marina Vannucci, editors, *Bayesian Inference for Gene Expression and Proteomics*, pages 177–201. Cambridge University Press, New York, NY, 2006.
